# Positive Cooperativity Induced by Interstrand Interactions in Silver(I) Complexes with α,α′‐Diimine Ligands

**DOI:** 10.1002/chem.202200912

**Published:** 2022-06-21

**Authors:** Davood Zare, Claude Piguet, Alessandro Prescimone, Catherine E. Housecroft, Edwin C. Constable

**Affiliations:** ^1^ Department of Chemistry University of Basel BPR 1096, Mattenstrasse 24a 4058 Basel Switzerland; ^2^ Department of Inorganic and Analytical Chemistry University of Geneva 30 quai E. Ansermet 1211 Geneva 4 Switzerland

**Keywords:** bipyridine, cooperativity, phenanthroline, silver, thermodynamics

## Abstract

The allosteric positive cooperativity accompanying the formation of compact [Cu^I^(α,α′‐diimine)_2_]^+^ building blocks contributed to the historically efficient synthesis of metal‐containing catenates and knotted assemblies. However, its limited magnitude can easily be overcome by the negative chelate cooperativity that controls the overall formation of related polymetallic multistranded helicates and grids. Despite the more abundant use of analogous dioxygen‐resistant [Ag^I^(α,α′‐diimine)_2_]^+^ units in modern entangled metallo‐supramolecular assemblies, a related thermodynamic justification was absent. Solid‐state structural characterizations show the successive formation of [Ag^I^(α,α′‐diimine)(CH_3_CN)][X] and [Ag^I^(α,α′‐diimine)_2_][X] upon the stepwise reactions of α,α′‐diimine=2,2′‐bipyridine (bpy) or 1,10‐phenanthroline (phen) derivatives with AgX (X=BF_4_^−^, ClO_4_^−^, PF_6_^−^). In room‐temperature, 5–10 mM acetonitrile solutions, these cationic complexes exist as mixtures in fast exchange on the NMR timescale. Spectrophotometric titrations using the unsubstituted bpy and phen ligands point to the statistical (=non‐cooperative) binding of two successive bidentate ligands around Ag^I^, a mechanism probably driven by the formation of hydrophobic belts, that overcomes the unfavorable decrease in the positive charge borne by the metallic cation. Surprisingly, the addition of methyl groups adjacent to the nitrogen donors (6,6′ positions in dmbpy; 2,9 positions in dmphen) induces positive cooperativity for the formation of [Ag(dmbpy)_2_]^+^ and [Ag(dmphen)_2_]^+^, a trend assigned to additional stabilizing interligand interactions. Adding rigid and polarizable phenyl side arms in [Ag(Brdmbpy)_2_]^+^ further reinforces the positively cooperative process, while limiting the overall decrease in metal–ligand affinity.

## Graphical abstract

**Big softie**: Ag^I^ ([Kr]4d^10^) possesses similar electronic structure to Cu^I^ ([Ar]3d^10^), but a larger ionic radius and increased covalency when bound to soft α,α′‐diimine ligands. Consequently, the thermodynamic formation of unsaturated [Ag^I^(α,α′‐diimine)]^+^ complexes is favored in organic solvents. Moreover, the addition of a second bidentate ligand to give saturated pseudo‐tetrahedral [Ag^I^(α,α′‐diimine)_2_]^+^ building blocks is driven by positive allosteric cooperativity when the ligands are decorated with terminal methyl groups.

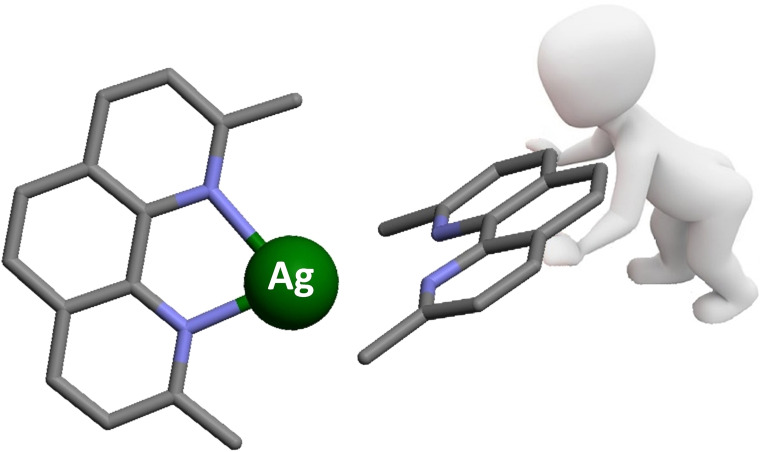

## Introduction

**Figure 1:**
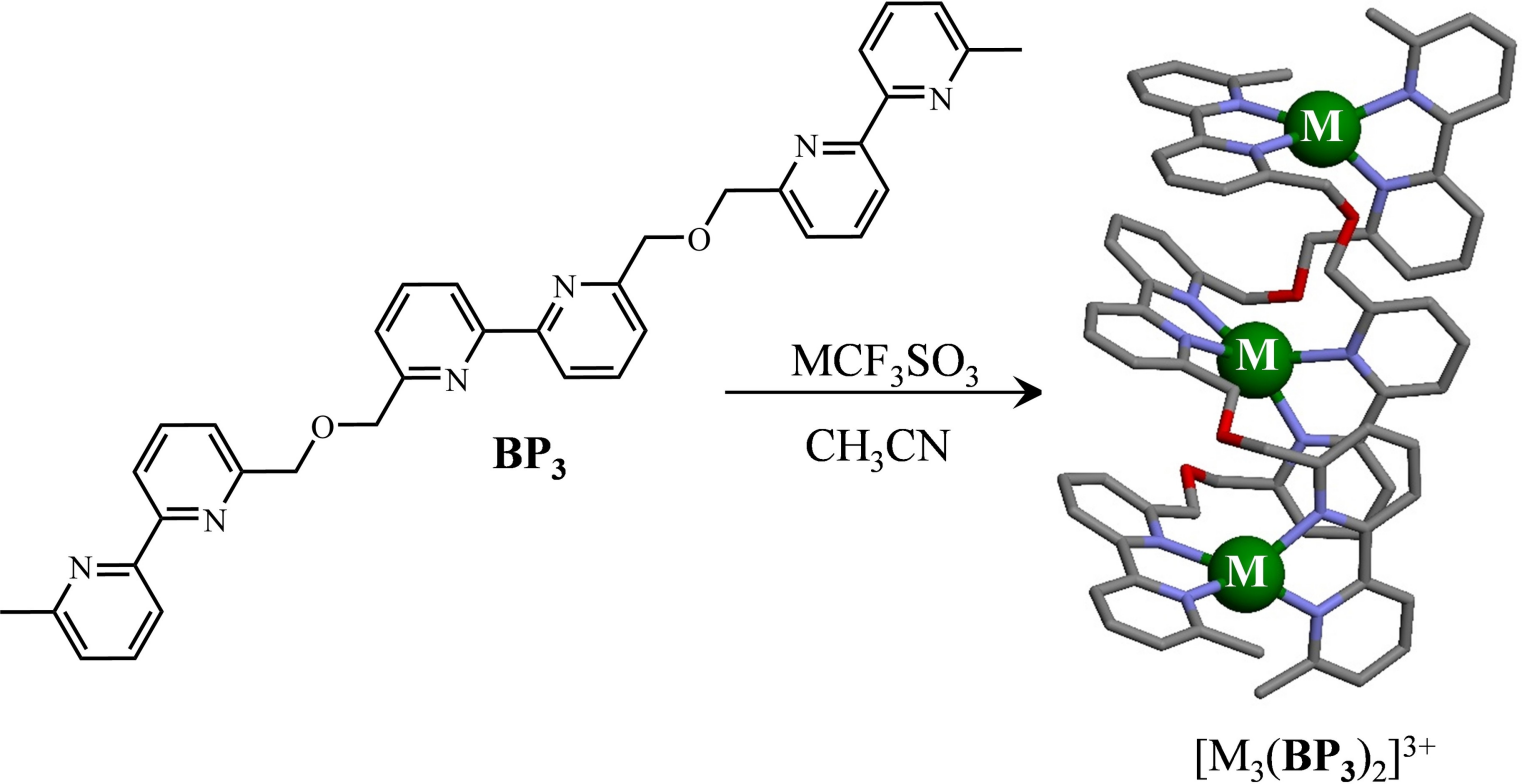
Self‐assembly of the double‐stranded helicates [M_3_(BP_3_)_2_]^3+^ (M=Cu^I[13]^ or Ag^I^;^[14]^ BP=bipyridine). The crystal structure of [Ag_3_(BP_3_)_2_](CF_3_SO_3_)_3_ is redrawn from refcode SEMLOL (CCDC‐1257126).^[14]^ Color code: C=gray, N=blue, O=red, Ag=green.

The systematic exploitation of metal–ligand coordination bonds for preparing sophisticated (supra)molecular architectures under thermodynamic control such as cages, grids and clusters,[^1^, ^2^, ^3^, ^4^] nanostructures[^5^, ^6^, ^7^, ^8^] or entangled rotaxanes, catenates and knots[^9^, ^10^, ^11^, ^12^] during the last three decades finds its origin in the seminal report of the spontaneous assembly of double‐stranded helicates from oligobipyridine ligands and copper(I)^[13]^ or silver(I)^[14]^ cations by Lehn and co‐workers (Figure 1).^[15]^

**Scheme 1:**
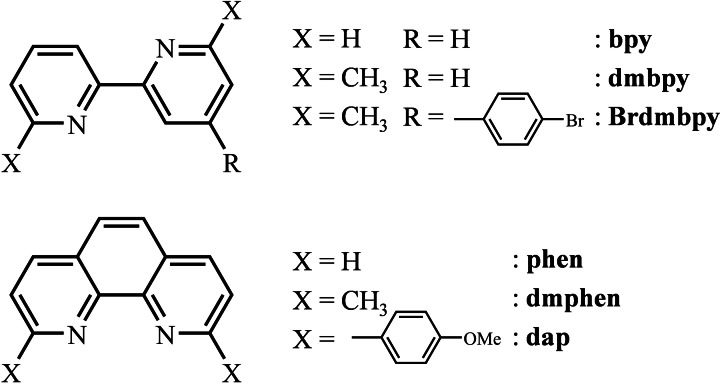
Chemical structures and acronyms used for the chelating α,α′‐diimine ligands considered in this work.

The diamagnetic character of these closed‐shell d^10^ cations greatly helped in the original characterization of their complexes in solution by NMR spectroscopic techniques, whereas their preferences for soft bidentate chelating ligands make them ideal for working as crucial partners in the design of multi‐diimine helicates,[^16^, ^17^, ^18^] grids, catenates and knots.[^12^, ^19^, ^20^, ^21^] With the extension of the binding possibilities provided by d‐block or f‐block cations with variable stereochemical preferences, the original Cu^I^‐ and Ag^I^‐based double‐stranded helicates were rapidly complemented with metal‐containing architectures possessing increased complexities; a part of supramolecular chemistry referred to as metallo‐supramolecular chemistry.[^22^, ^23^, ^24^, ^25^, ^26^] The sudden burst, during the early 1990s, of elaborate metallo‐supramolecular assemblies obtained by the straightforward mixing of segmental ligands with metal cations pointed to some unexpected and/or novel driving forces outside the standard concepts of coordination chemistry. The claim for positive cooperativity accompanying these self‐assembly process, as supported by some tentative building of thermodynamic Scatchard plots limited to intermolecular interactions,[^27^, ^28^] brought confidence that some favorable conditions helped in the formation of the desired polynuclear assemblies through the principle of “maximum site occupancy”, a phenomenon rarely invoked in standard coordination chemistry,^[29]^ but frequent in biology.[^30^, ^31^, ^32^, ^33^] In their seminal work investigating the mechanism of formation [Cu_3_(BP_3_‐COEt)_2_]^3+^ (a soluble derivative of [Cu_3_(BP_3_)_2_]^3+^), Albrecht‐Gary and co‐workers cast some reasonable doubts on the use of tools strictly limited to intermolecular connections for establishing global cooperativity.^[34]^ The highly welcome reminder by Ercolani in 2003 that intra‐ and intermolecular connection processes must be considered separately, restored a satisfying and pertinent modeling for the global thermodynamic self‐assembly of [Ag_3_(BP_3_)_2_]^3+^ and [Cu_3_(BP_3_‐COEt)_2_]^3+^,[^35^, ^36^] which finally appeared to be anti‐cooperative.^[37]^ In fact, the considerable energy costs accompanying the intramolecular processes (assigned to negative chelate cooperativity)[^38^, ^39^, ^40^] overcome some minor deviations from statistical behavior produced by the allosteric cooperativity accompanying the intermolecular processes.^[41]^ Focusing on the first steps of the complexation process where two α,α′‐diimine units of a ligand **L** (Scheme 1) are successively (intermolecularly) bound to Cu^I^ to give [Cu**L**]^+^ and [Cu**L**_2_]^+^ [Eqs. (1) and (2)], Albrecht‐Gary and co‐workers were indeed able to demonstrate that the two initial intermolecular associations were systematically driven by positive allosteric cooperativity (Appendix 1 in the Supporting Information).^[42]^

(1) Cu+solvated+L←→[CuL]+solvatedβCu,L1,1=ωCu,L1,1fLCu

(2) Cu+solvated+2L←→[CuL2]+solvatedβCu,L1,2=ωCu,L1,2(fLCu)2uL-LCu

Limited to the standard diimine ligands L=2,2′‐bipyridine (bpy), dmbpy (dm=dimethyl), 1,10‐phenanthroline (phen), dmphen, the latter favorable deviations from statistics amount to −3≤ΔEL-LCu =−*RT* ln(uL-LCu )≤−1.1 kJ mol^−1^ and was considered as an important driving force for the formation of both helicates and catenates with this metal. Moreover, the striking increase by one order of magnitude of the positive cooperativity accompanying the fixation of a second ligand to give [Cu**L**_2_]^+^ in going from (2,9‐dimethyl)phenanthroline in [Cu(dmphen)_2_]^+^ (ΔEL-LCu =−1.0 kJ mol^−1^) to [Cu(dap)_2_]^+^ (ΔEL-LCu =−13.6 kJ mol^−1^) was assigned to improved intramolecular interligand face‐to‐face π–π stacking interactions produced by the compact orthogonal organization of the two ligands around the small Cu^I^ center.^[42]^ Surprisingly, the latter comprehensive thermodynamic analysis was strictly limited to the binding of the oxidation‐sensitive Cu^I^ to α,α′‐diimine ligands, whereas the larger 4d^10^ analogue, Ag^I^, escaped such a detailed and rational analysis. Only partial energy changes and driving forces have been discussed for silver helicates,[^27^, ^43^, ^44^] cages,^[45]^ catenates,^[46]^ and grids,^[47]^ despite the extensive use of Ag^I^ partners for preparing complicated metal‐peptide strands^[7]^ and self‐assembled aggregates with catalytic,^[48]^ optical^[49]^ and biomedical applications.[^50^, ^51^, ^52^] In this work, we aim at filling the gap between the well‐known thermodynamics for the formation of the pseudo‐tetrahedral [Cu^I^(N^∩^N)_2_]^+^ building blocks and the mainly ignored processes leading to the oxidatively‐robust [Ag^I^(N^∩^N)_2_]^+^ analogues where N^∩^N is a bidentate α,α′‐diimine ligand.

## Results and Discussions

### Synthesis and crystal structures of [AgL_2_][X] complexes with L=bpy, dmbpy, Brdmbpy, phen, dmphen (X=ClO^−^, BF_4_^−^, PF_6_^−^)

**Figure 2:**
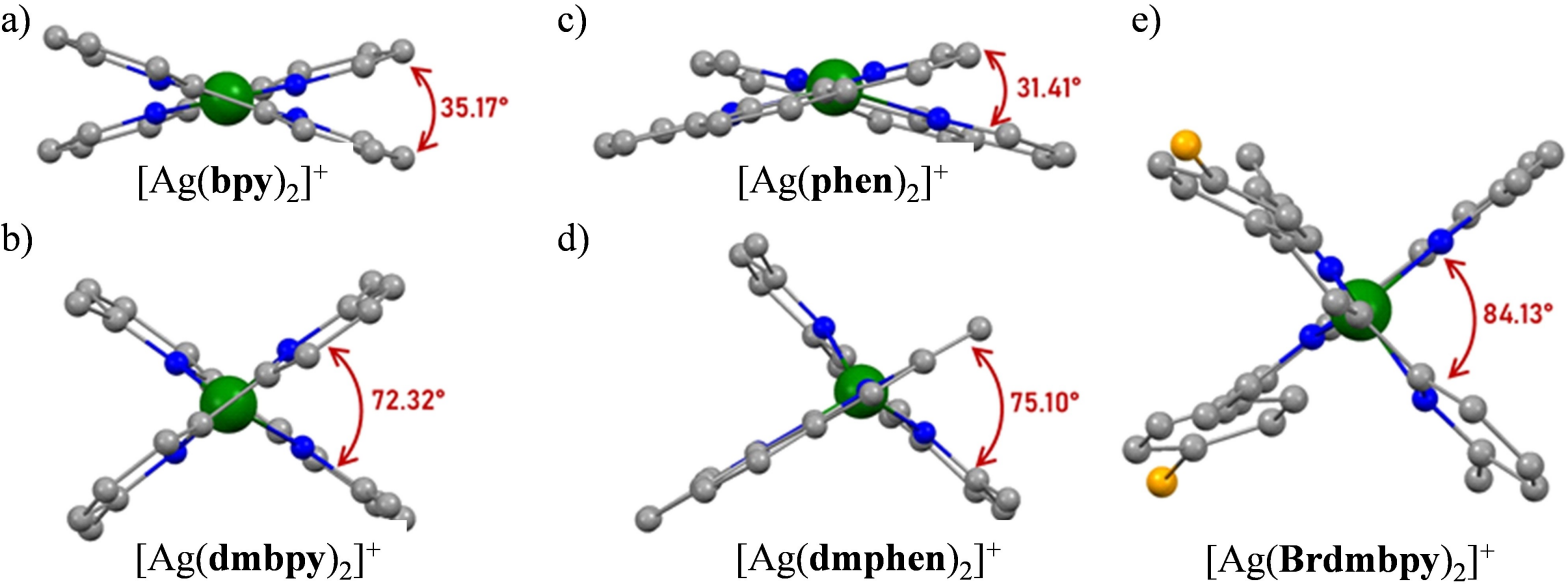
Molecular structures of the [Ag**L**_2_]^+^ cations observed in the crystal structures of a) [Ag(bpy)_2_][ClO_4_] (refcode MAYCIZ; CCDC‐260789),^[57]^ b) [Ag(dmbpy)_2_][BF_4_] (refcode UFABAF; CCDC‐657584),^[58]^ c) [Ag(phen)_2_][ClO_4_] (refcode MAYCOF; CCDC‐260790),^[57]^ d) [Ag(dmphen)_2_][PF_6_], and e) [Ag(Brdmpby)_2_][PF_6_]. Color code: C=gray, N=blue, Ag=green, Br=orange.

Among the bidentate α,α′‐diimine ligands investigated for their thermodynamic stabilities with Cu^I^ in organic solvents (Scheme S1), commercially available bpy, dmbpy, phen and dmphen have been selected for analogous studies with Ag^I^ in order to explore the effects of i) rigid preorganization (bpy vs. phen or dmbpy vs. dmphen) and ii) interligand interactions (bpy vs. dmbpy or phen vs. dmphen) on the structures and stability constants of the pseudo‐tetrahedral [Ag**L**_2_]^+^ complexes (Scheme 1). Additionally, the extended ligand Brdmbpy ligand was prepared (Scheme S2)^[53]^ for testing potential effects of electronic delocalization accompanying the connection of rigid aromatic groups for the linking of [Ag**L**_2_]^+^ building blocks to polymeric backbones or surfaces. A rational control of this effect is crucial for further applications, since it has been recognized as the vector which limits the affinity of binding sites in metallopolymers made of analogous terdentate binding units connected by phenyl rings.[^54^, ^55^, ^56^] Stoichiometric reactions of the bidentate bpy, phen or dmbpy ligands with silver(I) salts in polar organic solvents have been shown[^57^, ^58^, ^59^] to give 1 : 1 [Ag**L**][X] and 1 : 2 [Ag**L**_2_][X] complexes (X=ClO_4_^−^, BF_4_^−^, PF_6_^−^), the reported crystal structures of which are shown in Figure 2a–c together with pertinent geometrical parameters in Table 1 (entries 1–4). Repeating here the same process for **L**=dmphen and Brdmbpy with AgPF_6_ in acetonitrile provided [Ag(dmphen)_2_][PF_6_] (Figures 2d and S1, Tables S2–S4), [Ag(Brdmpby)_2_][PF_6_] (Figures 2e and S2, Tables S5–S7) and [Ag(Brdmpby)(CH_3_CN)][PF_6_] (Figure S3 and Tables S8–S10).

**Table 1:** Average bond lengths, bite angles and total distortion from octahedral geometry *∑* ^[a]^ observed in the crystal structures of [Ag(bpy)_2_][ClO_4_], [Ag(phen)_2_][ClO_4_], [Ag(dmbpy)_2_][BF_4_], [Ag(dmphen)_2_][PF_6_] and [Ag(Brdmbpy)_2_][PF_6_].^[b]^

Complex	Ag−N distance [Å]	Chelate N−Ag−N angle [°]	Interligand dihedral angle [°]	*∑* [°]	Ref.
[Ag(bpy)_2_]^+^	2.33(1)	71.46(5)	26.37	189.4	[57]
[Ag(phen)_2_]^+^	2.34(8)	71.76(5)	32.85	183.8	[57]
[Ag(phen)_2_]^+^	2.33(5)	72.20(4)	29.37	185.8	[57]
[Ag(dmbpy)_2_]^+^	2.30(1)	72.48(5)	75.32	158.4	[58]
[Ag(dmphen)_2_]^+^	2.33(7)	72.31(1)	75.10	161.1	this work
[Ag(Brdmbpy)_2_]^+^	2.31(3)	72.3(3)	84.13	157.6	this work

[a] ∑ is calculated with the formula ∑=$\overset{6}{\underset{i=1}{\sum }}$ |109.5−*f*_i_| where *f*_i_ are the 6 bond angles around the Ag^I^ metal. [b] The values given between parentheses correspond to the standard deviation when several distances or bond angles are considered.

In the solid state, all isolated salts with Ag : L=1 : 2 stoichiometry exist as cationic silver complexes [Ag**L**_2_]^+^ accompanied by non‐interacting ionic counter‐anions. The Ag−N bond lengths cover the 2.30–2.40 Å range as previously found for related [Ag^I^(N^∩^N)_2_]^+^ cations obtained with polysubstituted bpy and phen ligands.^[60]^ Compared with the corresponding the [CuN_4_]^+^ units ([Ar]3d^10^ configuration, average Cu−N bond distance=2.03 Å),[^60^, ^61^] the Ag−N distances are on the average 0.3 Å longer, while an increase of 0.40 Å is expected on the basis of the 4‐coordinate ionic radii of Cu^+^ and Ag^+^.^[62]^ However, Δ(M−L)=*d*(Ag^I^‐L)−*d*(Cu^I^‐L) tends to decrease when covalent bonding forces are important, a trend expected with these relatively soft and polarizable α,α′‐diimine ligands.^,[63][64]^ Within the series of tetracoordinate [Ag^I^(N^∩^N)_2_]^+^ complex cations considered in Figure 2, the change in interligand dihedral angle is the only striking geometrical variation. For unsubstituted bpy and phen ligands, the flattening is ascribed to lattice effects that can be traced back to intermolecular interactions involving the heteroaromatic ligands.^[60]^ The connection of methyl groups to the 6,6′‐position of the α,α′‐diimine skeleton favors larger dihedral angles while maximizing local steric crowding to give values in the range 75–85° for the [Ag(dmbpy)_2_]^+^, [Ag(dmphen)_2_]^+^ and [Ag(Brdmbpy)_2_]^+^ cations. A previous thorough structural analysis of the molecular structures of [Cu(4,4′‐dimethyl‐dmbpy)_2_]^+^ and [Ag(4,4′‐dimethyl‐dmbpy)_2_]^+^ ruled out the possibility that the flattening was due to admixture of charge‐transfer excited state configurations and it ascribed the variable interligand dihedral angles to lattice effects.^[60]^

Complexes [Ag**L**]^+^ with a 1 : 1 metal/ligand stoichiometric ratio could be isolated in the solid state following the mixing of Brdmbpy (1.0 equiv.) with AgPF_6_ (1.0 equiv.). The isolated single crystals display a roughly planar three‐coordinated complex cation in [Ag(Brdmpby)(CH_3_CN)][PF_6_] (Figure S3), in which the two Ag−N(pyridine) bond distances (2.2852(15) and 2.2658(16) Å, Table S8) are on the lower limit of those found for the related [AgN_4_]^+^ units.

### Stabilities, speciations and structures of [AgL_*n*_]^+^ complexes in acetonitrile solution (L=bpy, dmbpy, Brdmbpy, phen, dmphen)

**Figure 3:**
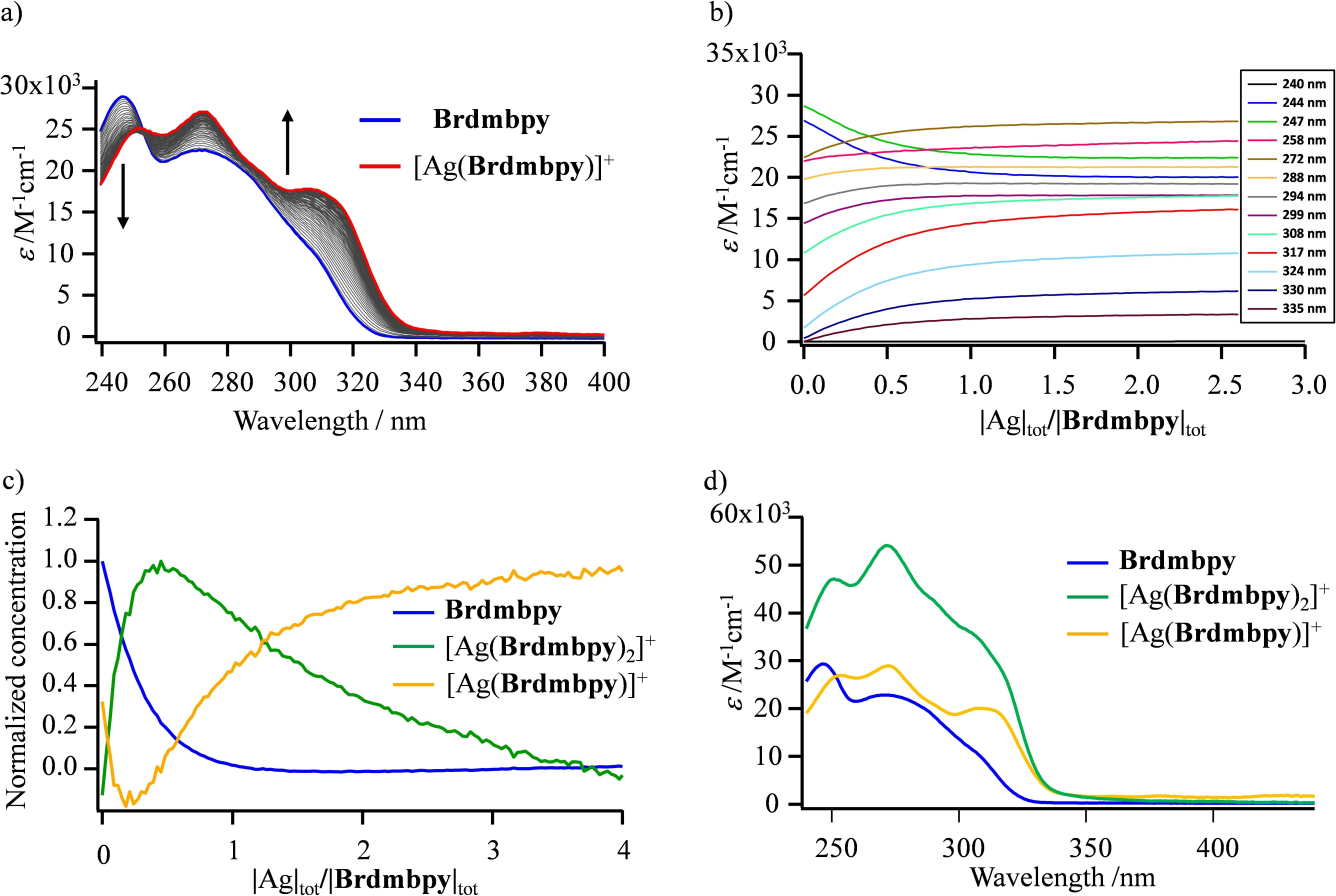
a) Variation of experimental absorption spectra and b) corresponding variation of molar extinction at different wavelengths observed for the spectrophotometric titration of Brdmbpy with AgPF_6_ (total ligand concentration: 2.3×10^−4 M^ in acetonitrile, 298 K, titration increment |Ag|_tot_/|Brdmbpy|_tot_≈0.05). c) Evolving factor analysis[^68^, ^69^, ^70^, ^71^] using three absorbing eigenvectors, each normalized to a maximum concentration of 1.0^[73]^ and d) reconstructed individual electronic absorption spectra.

The transformations of the UV absorption spectrum, produced by the structural reorganization and the changes in polarization accompanying the complexation of bidentate α,α′‐diimine units to metallic cations,[^65^, ^66^, ^67^] have been used to extract thermodynamic data with Cu^I^ salts (Table 2, entries 6–9)[^32^, ^33^, ^34^, ^42^] by principal component analyses of the spectrophotometric titration data.[^68^, ^69^, ^70^, ^71^, ^72^, ^73^] As it is well established that Cu^I^ and Ag^I^ cations react similarly with α,α′‐diimine units according to Equilibria (1) and (2),[^57^, ^60^] pertinent thermodynamic stability constants could be obtained by using the same model applied to the spectrophotometric titrations of **L** with AgPF_6_ in acetonitrile (Figures 3a, b and S4a,b–S7a,b). Evolving factor analyses[^68^, ^69^, ^70^, ^71^] confirmed the existence of three absorbing species (Figures 3c and S4c–S7c), while nonlinear least‐square fits[^72^, ^73^] to Equilibria (1) and (2) provided stability constants (Table 2, entries 1–5) together with satisfying reconstructed absorption spectra for the pure species **L**, [Ag**L**]^+^ and [Ag**L**_2_]^+^ (Figures 3d and S4d–S7d).

**Table 2:** Thermodynamic formation constants, associated microscopic parameters ΔGM,Laff and ΔEL-LM and computed speciations obtained for the spectrophotometric titrations of L with AgPF_6_ or CuBF_4_ at 298 K.^[a]^

Ligand	Metal	log (βM,L1,1 )	log (βM,L1,2 )	ΔGM,Laff	ΔEL-LM	Solvent	Speciation^[c]^				Ref.
				[kJ mol^−1^]	[kJ mol^−1^]		|M|_tot_/|L|_tot_=1		|M|_tot_/|L|_tot_=0.5		
							|ML| [%]	|ML_2_| [%]	|ML| [%]	|ML_2_| [%]	
bpy	Ag^I^	5.94(5)	10.74(5)	−27.7(1)	0.3(1)	MeCN	65	35	3	95	this work
dmbpy	Ag^I^	4.71(5)	9.12(5)	−20.7(1)	−4.4(1)	MeCN	41	58	4	92	this work
Brdmbpy	Ag^I^	4.44(5)	8.73(6)	−19.2(1)	−5.3(1)	MeCN	37	62	5	90	this work
phen	Ag^I^	6.11(5)	11.11(5)	−28.7(1)	0.3(1)	MeCN	64	36	2	96	this work
dmphen	Ag^I^	5.83(5)	11.31(5)	−27.1(1)	−4.1(1)	MeCN	43	57	1	98	this work
bpy	Cu^I^	3.69(8)	6.5(1)	−14.9(3)	−1.1(1)	MeCN^[d]^	57	34	18	58	[34]
dmbpy	Cu^I^	5.4(2)	10.2(3)	−24(1)	−3.0(2)	MeCN^[d]^	50	50	3	95	[34]
phen	Cu^I^	5.2(1)	9.7(1)	−23.5(4)	−2.2(5)	MeCN^[d]^	47	53	4	92	[42]
dmphen	Cu^I^	6.6(2)	12.3(3)	−31.5(6)	−1.0(1)	MeCN^[d]^	41	58	1	98	[42]

[a] The site binding model used βM,L1,1 =12 fLM and βM,L1,2 =12 (fLM )^2^ uL-LM ) for all ligands. [b] ΔGM,Laff =−*RT* ln(fLM ) and ΔEL-Laff =−*RT* ln(uL-LM ). [c] Ligand speciation computed with Equilibria (1) and (2) for |Ag|_tot_=5×10^−3^ M in acetonitrile. ^[d]^ MeCN+0.1 M NEt_4_ClO_4_.

Although the ionic radius of tetra‐coordinate Ag^I^ is 0.4 Å larger than Cu^I^,^[62]^ their affinities for the selected bidentate α,α′‐diimine ligands, as estimated by log(βM,L1,1 ), are comparable (Table 2) with even a slightly greater affinity for Ag^I^, assuming that the addition of the inert electrolyte NEt_4_ClO_4_ (Table 2, entries 6–9) has limited influence on the complexation of neutral ligands to the weakly charged cationic centers. Taking for granted that solvated 1 : 1 [Ag**L**]^+^ complexes exist in solution as planar three‐coordinate [Ag**L**(CH_3_CN)]^+^ units, as found in the crystal structure of [Ag**(**Brdmbpy**)**(CH_3_CN)][PF_6_] (Figure S3), the computed statistical factors ωAg,L1,1 =12 and ωAg,L1,2 =12 (Appendix 3) match those found for four‐coordinate [Cu(**L**)(CH_3_CN)_2_]^+^ (Appendix 2). Within the context of the site binding model,[^74^, ^75^, ^76^] Equations (3) and (4) are thus pertinent for extracting metal–ligand affinities ΔGM,Laff =−*RT* ln(fLM ) (Table 2, column 5) together with interligand interactions ΔEL-LM =−*RT* ln(uL-LM ) (Table 2, column 6) for both Cu^I^ and Ag^I^.

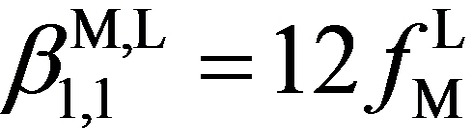




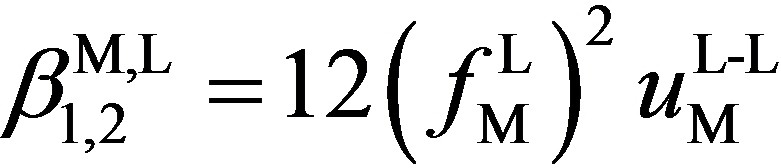




Firstly, the metal–ligand affinities −28.7≤ΔGAg,Laff ≤−19.2 kJ ⋅ mol^−1^ (Table 2, entries 1–5) compare well with those of Cu^I^ with the same ligands (−31.5≤ΔGCu,Laff ≤−14.9 kJ ⋅ mol^−1^, Table 2, entries 6–9) because the larger covalency between the extended metal‐centered 5 s and 5p orbitals with the ligand‐based orbitals compensates for the larger Ag^I^ ionic radius and the decrease of the electrostatic contribution.[^60^, ^63^, ^77^] Secondly, the expected ligand‐based preorganization effect is confirmed with i) ΔGM,bpyaff being less favorable than ΔGM,phenaff and ii) ΔGM,dmbpyaff being less favorable than ΔGM,dmphenaff . One notes that the latter trend seems less pronounced with Ag^I^, probably because the preference of this large cation for five‐membered rings (instead of the preference of small Cu^I^ cations for six‐membered rings)[^78^, ^79^] does not require specific structural constraints in the final chelate rings with α,α′‐diimine units, which makes Ag^I^ less sensitive to preorganization.

Thirdly, the attachment of methyl groups close to the metallic center in dmbpy (6,6′‐positions) and dmphen (2,9‐positions) reinforces the affinity for the entering bidentate α,α′‐diimine unit around the smallest Cu^I^ cation with (ΔGCu,Laff −ΔGCu,dmLaff )=8 to 10 kJ mol^−1^, which can be rationalized by favorable inductive or charge transfer effects.^[80]^ The reverse situation occurs with the larger Ag^I^ center with (ΔGAg,Laff −ΔGAg,dmLaff )=−2 to −7 kJ mol^−1^; a trend tentatively assigned to steric hindrance for the entering metal compensating for the increased methyl‐based inductive effects. Finally, the connection of extra electro‐deficient phenyl rings to the polyaromatic diimine unit in [Ag**(**Brdmbpy)_2_]^+^ has only minor effects of the Ag–ligand affinity, which is reduced by 5 % compared with [Ag(dmbpy)_2_]^+^, a value still compatible with the use of this building block for the design of metallopolymers.[^54^, ^55^, ^56^]

**Figure 4:**
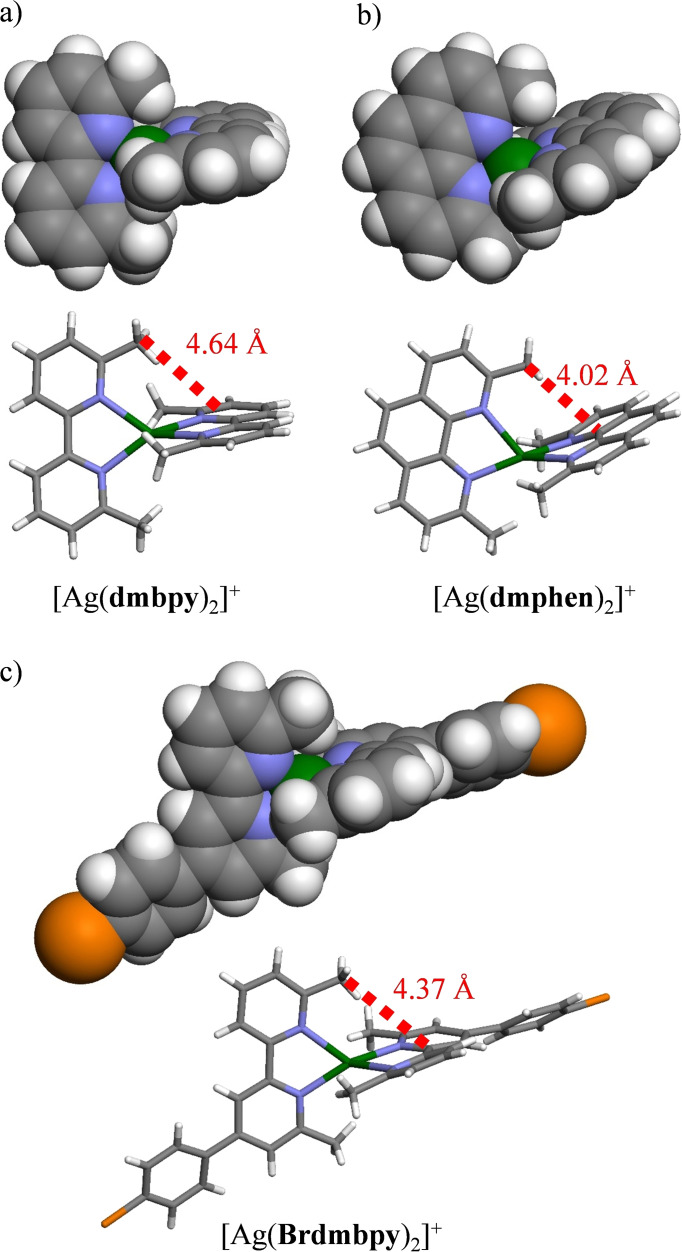
Space‐filling model (CPK)^[81]^ showing the close interligand H_3_C⋅⋅⋅aromatic interactions as found in the crystal structures of a) [Ag(dmbpy)_2_][BF_4_] (refcode UFABAF; CCDC‐657584)_,_ ^[58]^ b) Ag(dmphen)_2_][PF_6_] and c) [Ag(Brdmpby)_2_][PF_6_]. Color codes: C=gray, N=blue, Ag=green, Br=orange. The distance between the C(methyl) and the middle of the C−C(diimine) bond is highlighted.

Focusing our attention on the interligand interactions, ΔEL-LM for L=bpy, dmbpy, phen, dmphen reveals a rather constant and weak (|ΔEL-LM |≤*RT*≈2.5 kJ ⋅ mol^−1^) positive cooperativity for the fixation of the second ligand to Cu^I^ to give [Cu**L**_2_]^+^ (−3.0≤ΔEL-LCu ≤−1.0 kJ ⋅ mol^−1^, Table 2 column 6). Again, the situation changes with the larger Ag^I^ metallic cation since the parent bpy and phen ligands follow statistical binding behavior (ΔEL-LAg ≈ 0 kJ ⋅ mol^−1^), while the more constrained dmbpy and dmphen ligands exhibit noticeable positively cooperative mechanisms (−4.4≤ΔEL-LAg ≤−4.1 kJ ⋅ mol^−1^, Table 2 column 6) with a maximum drift for the most polarizable Brdmbpy ligand (ΔEL-LAg =−5.3 kJ ⋅ mol^−1^). A careful look at the molecular structures of [Ag(dmbpy)_2_]^+^, [Ag(dmphen)_2_]^+^ and [Ag(Brdmbpy)_2_]^+^ with 6,6′‐dimethyl substituted ligands suggests some extra stabilization through van der Waals interactions involving the methyl groups of one ligand, which are located close to the polyaromatic rings of the second bound ligand (Figure 4).

**Figure 5:**
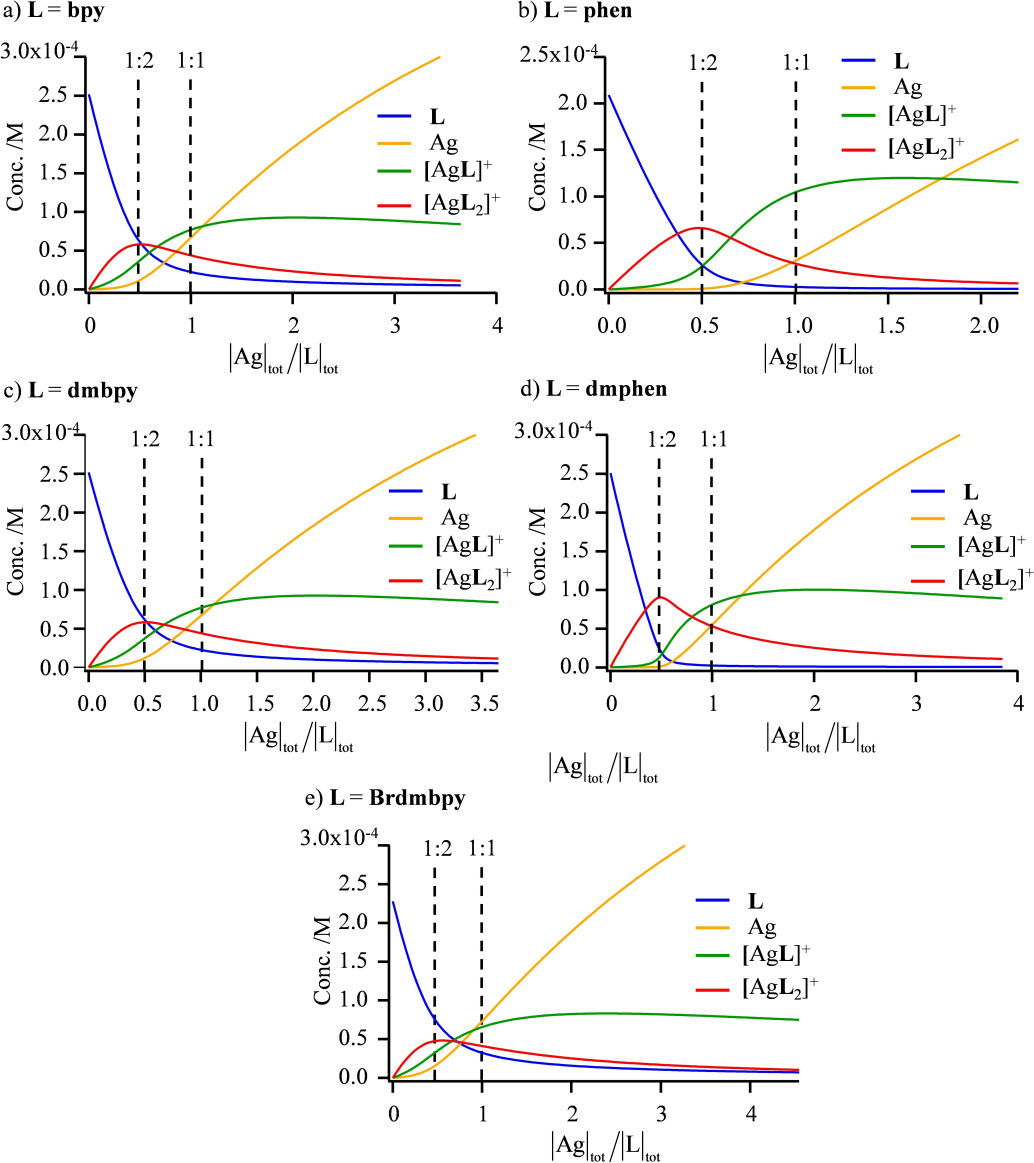
Computed speciations computed for the spectrophotometric titrations of a) bpy (|L|_tot_=2.81×10^−4^ M), b) dmbpy (|L|_tot_=2.09×10^−4^ M), c) phen (|L|_tot_=2.52×10^−4^ M), d) dmphen (|L|_tot_=2.51×10^−4^ M) and e) Brdmbpy (|L|_tot_=2.30×10^−4^ M) with a solution of AgPF_6_ in acetonitrile.

However, the deviations from statistical binding still remain limited for both Cu^I^ and Ag^I^ complexes with α,α′‐diimine ligands and the computed speciation accompanying the spectrophotometric data recorded at |L|_tot_=2×10^−4^ M points to significant mixtures of **L**, [Ag**L**]^+^ and [Ag**L**_2_]^+^ for the target stoichiometric |Ag|_tot_/|L|_tot_=1 and |Ag|_tot_/|L|_tot_=0.5 ratios (Figure 5). It is useful to stress here that the latter stoichiometric ratios correspond to the mixtures obtained when solid state [Ag**L**][PF_6_] and [Ag**L**_2_][PF_6_] complexes are dissolved in acetonitrile (Figure 5). According to the law of mass action (Le Chatelier's principle), a global increase in |L|_tot_ narrows the distributions of the various species in solution, but straightforward calculations still predict that, for |Ag|_tot_=|L|_tot_=5×10^−3^ M (=5 mM) used for the NMR spectroscopic studies performed in CD_3_CN, more than one third (i. e., 33 %) of the ligand distribution exists as [Ag**L**_2_]^+^, whereas [Ag**L**]^+^ counts for less than two thirds (Table 2, column 8). On the contrary, a close‐to‐quantitative formation of the target [Ag**L**_2_]^+^ complex is expected for |Ag|_tot_/|L|_tot_=0.5 and |L|_tot_=1×10^−2^ M (=10 mM; Table 2, column 9).

**Figure 6:**
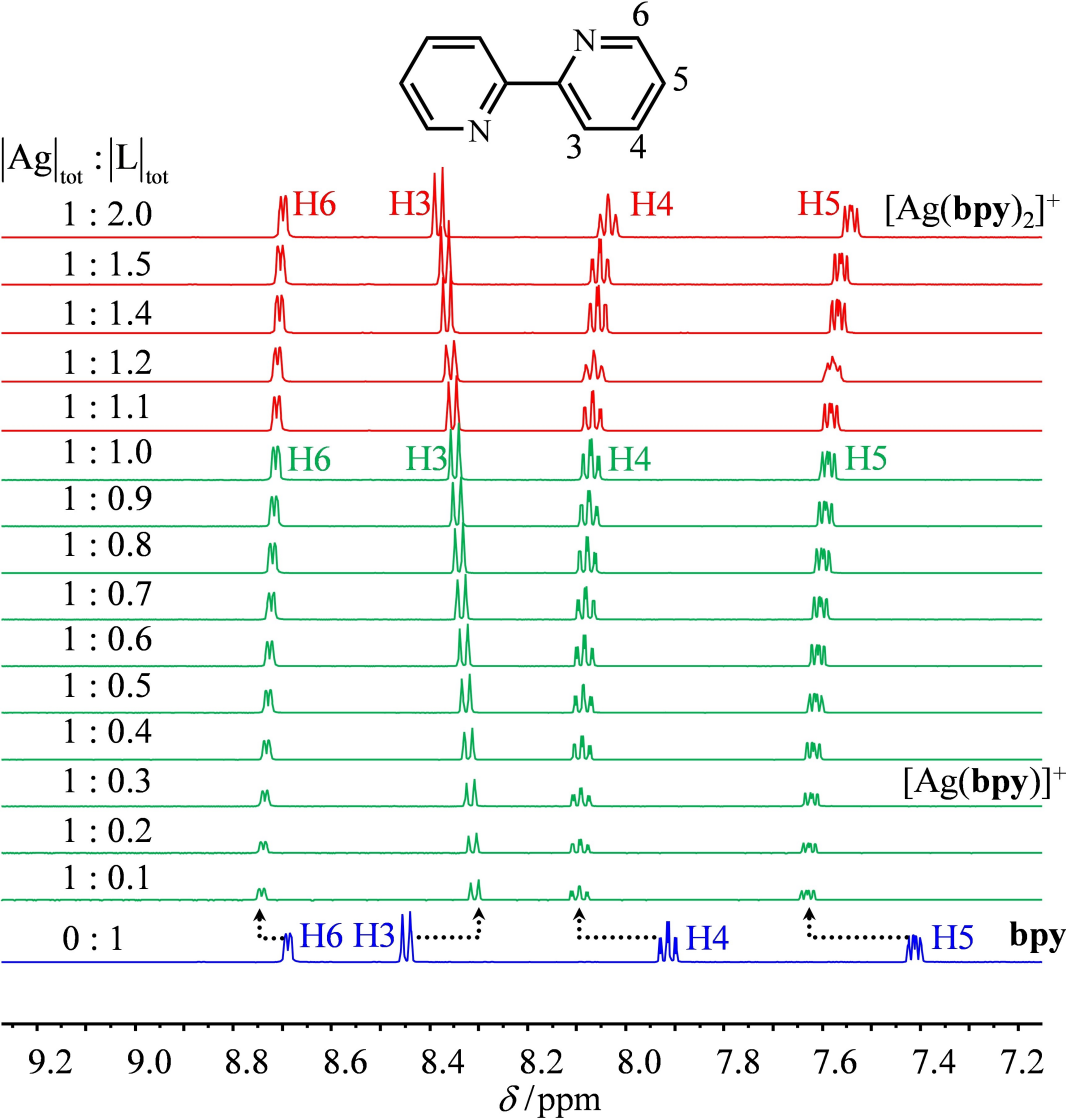
^1^H NMR batch titration of bpy (|L|_tot_ **=**5 mM) with AgPF_6_ (500 MHz, CD_3_CN, 293 K).

**Figure 7:**
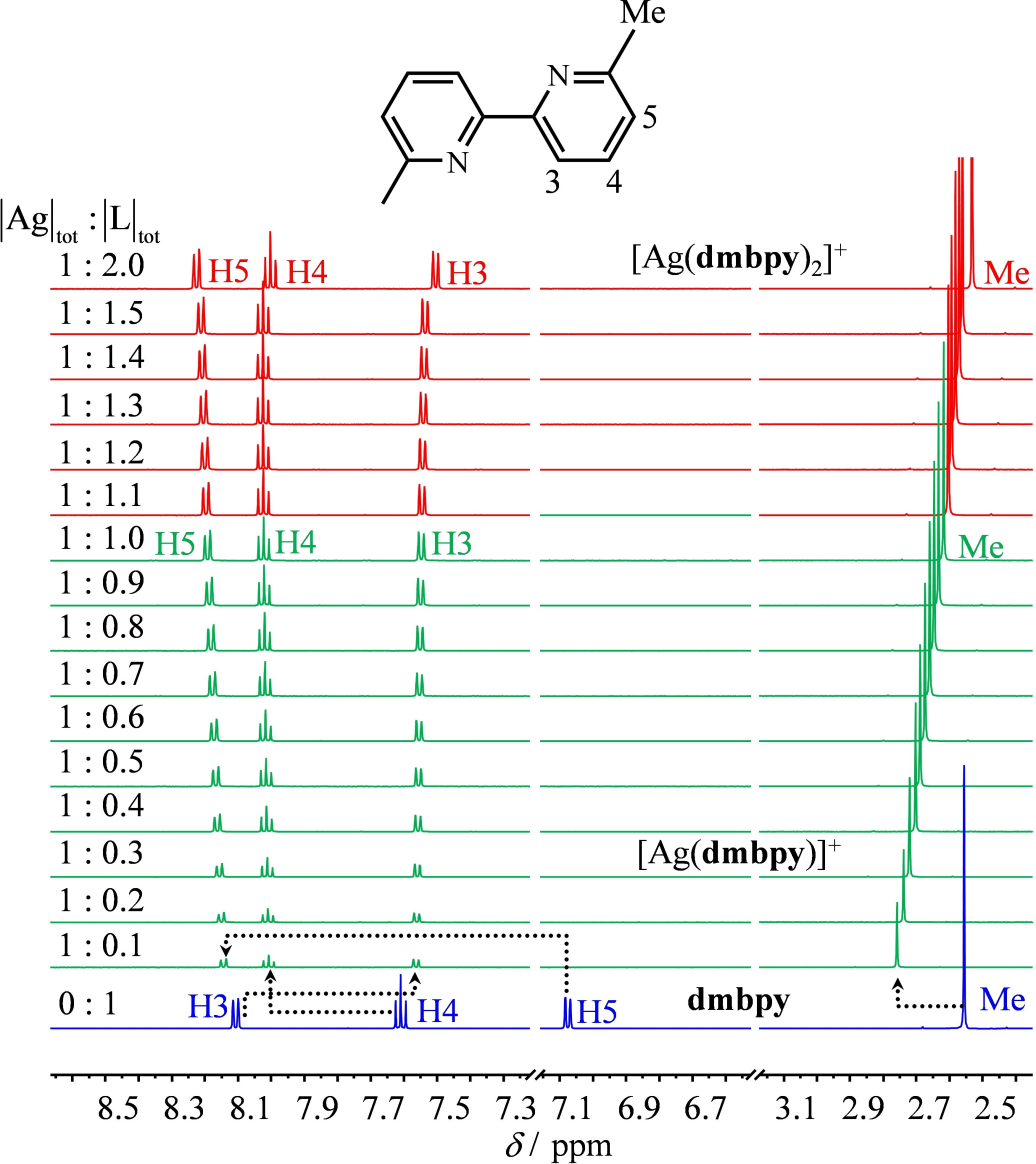
^1^H NMR batch titration of dmbpy (|L|_tot_=5 mM) with AgPF_6_ (500 MHz, CD_3_CN, 293 K).

The ^1^H NMR titrations of **L** with AgPF_6_ in CD_3_CN recorded for |L|_tot_=5×10^−3^ M at room temperature display dynamically‐average spectra, which are diagnostic for fast exchange processes operating between the ligand and its silver complexes on the NMR timescale (Figures 6, 7, S8, and S9). Combined with the speciation collected in Table 2 (columns 8 and 9), the ^1^H NMR spectra pertinent to [Ag**L**_2_]^+^ are indeed found for |Ag|_tot_/|L|_tot_=0.5, but the spectroscopic signatures of [Ag**L**]^+^ require a large excess of silver (|Ag|_tot_/|L|_tot_≥2; Figures 6, 7, S8, and S9). For the aromatic protons H4–H6 of the 2,2′‐bipyridine skeletons in bpy (Figure 6) and dmbpy (Figure 7), their complexation to Ag^+^ is accompanied by a global downfield shift due to polarization effects. The proton H3 is special in this context because its chemical shift is sensitive to the *syn*/*anti* conformation of the α,α′‐diimine unit. In the free ligand, the electronic density around H3 is reduced by the adjacent attractive nitrogen lone pair (*anti* conformation=downfield shift). The *anti*→*syn* conformational change accompanying the complexation of the bpy or dmbpy units to Ag^+^ removes this interaction and the signal of H3 is shifted upfield in the resulting [Ag**L**]^+^ and [Ag**L**_2_]^+^ complexes (Figures 6 and 7).

Finally, The ^1^H NMR signal of the methyl groups attached to the 6,6′‐positions of the bipyridine skeleton in dmbpy is initially downshifted due to polarization effects accompanying the complexation in [Ag(dmbpy)]^+^. The subsequent orthogonal fixation of the second ligand in [Ag(dmbpy)_2_]^+^ induces a stepwise upfield shift (Figure 7), which results from the location of the methyl groups of one ligand above the shielding cone of the polyaromatic bipyridine unit of the second ligand (Figure 4a). Very similar behaviors are observed for the related phen/dmphen ligand pair (Figures S8 and S9).

## Conclusions

As previously established for Cu^I^ complexes with α,α′‐diimine ligands, the successive binding of these bidentate units to Ag^I^ gives [Ag**L**_2_]^+^ with negligible‐to‐weak positive cooperativity. This trend contrasts with standard coordination chemistry in which the successive binding of electron‐rich neutral ligands to a cationic metallic center is usually accompanied by negative allosteric cooperativity.[^29^, ^76^, ^82^] For unsubstituted 2,2′‐bipyridine and 1,10‐phenanthroline backbones, as found in bpy and phen, the easy attachment of the second ligand in [M**L**_2_]^+^ (M=Cu^I^, Ag^I^) can be traced back to a favorable driving force produced by the formation of a hydrophobic belt in polar solvents,[^83^, ^84^, ^85^] a phenomenon responsible for the entropic stabilization of hydrophobic complexes in water.[^86^, ^87^] Although less impressive than the π–π intramolecular interligand stacking interactions previously demonstrated to be responsible for the positive cooperativity that occurs in [Cu(dap)_2_]^+^ (ΔEL-LCu ≤−10 kJ ⋅ mol^−1^; Table S1),^[42]^ extension of the diimine skeletons with methyl groups produces interstrand methyl–aromatic interactions in [Ag**L**_2_]^+^ (**L**=dmbpy, dmphen, Brdmbpy) that are not negligible (ΔEL-LAg ≤−5 kJ ⋅ mol^−1^, Table 2) and contribute to the successful use of [Ag^I^(N^∩^N)_2_]^+^ as a building block in coordination and metallo‐supramolecular chemistry. A retrospective look at the formation of double‐stranded [Cu_3_(BP_3_)_2_]^3+^ and [Ag_3_(BP_3_)_2_]^3+^ helicates confirms that the choice of the compact and constrained pseudo‐tetrahedral [M^I^(N^∩^N)_2_]^+^ building blocks (M=Cu, Ag) provides positive allosteric cooperativity for their formation, even if concomitant negative chelate cooperativity overcomes this initial favorable thermodynamic driving force.

## Experimental Section

The ligands **b**py, phen, dmbpy, dmphen and chemicals were purchased from Sigma‐Aldrich and were used without further purification unless otherwise stated. Brdmbpy was synthesized following a slightly modified version of the original procedure.^[53]^ The complexes [Ag(bpy)_2_][ClO_4_],^[57]^ [Ag(phen)_2_][ClO_4_]^[57]^ and [Ag(dmbpy)_2_][BF_4_]^[58]^ were prepared according to literature procedures.

***CAUTION***! Dry perchlorates can explode and should be handled in small quantities and with the necessary precautions.[^88^, ^89^]

Preparation of (*E*)‐3‐(4‐bromophenyl)‐1‐(6‐methylpyridin‐2‐yl)prop‐2‐en‐1‐one:^[53]^ 4‐Bromobenzaldehyde (3.35 g, 18.1 mmol, 1.0 equiv.) and 6‐methyl‐2‐acetylpyridine (2.47 g, 18.1 mmol, 1.0 equiv.) were dissolved in methanol (81.0 mL). Sodium hydroxide (17.0 mL, aqueous 2 %wt) was added, whereupon the solution turned green. The reaction mixture was left to stir at room temperature for 4 h. The white precipitate that formed was collected by filtration and washed carefully with water and methanol (3 x 20 mL each). The resulting light green solid was dried in a desiccator for 12 h to give (*E*)‐3‐(4‐bromophenyl)‐1‐(6‐methylpyridin‐2‐yl)prop‐2‐en‐1‐one (4.70 g, 15.5 mmol, 85 %). ^1^H NMR (CDCl_3_, 500 MHz): *δ*=8.33 (dd, *J*=16.2, 1.4 Hz, 1H), 8.00 (d, *J*=7.7 Hz, 1H), 7.86 (dd, *J*=16.0, 1.3 Hz, 1H), 7.77 (td, *J*=7.8, 1.4 Hz, 1H), 7.65–7.53 (m, 4H), 7.37 (d, *J*=7.6 Hz, 1H), 2.69 ppm (d, *J*=1.4 Hz, 3H)

Preparation of 1‐(2‐oxopropyl)pyridinium chloride:^[53]^ Pyridine (7.85 g, 8.02 ml, 99.2 mmol, 1.0 equiv.) was dissolved in THF (20 mL). Chloroacetone (11.0 g, 9.48 ml, 119 mmol, 1.2 equiv.) was added to the solution, which was then stirred for 48 h at room temperature. The resulting white precipitate was filtered, washed with THF and dried under high vacuum to give 1‐(2‐oxopropyl)pyridinium chloride (6.6 g, 48.9 mmol, 49 %) as a creamy white powder. The crude material was used without purification for the following reaction. ^1^H NMR ([D_6_]DMSO, 500 MHz): *δ*=9.07 (dd, *J*=6.6, 1.3 Hz, 2H), 8.66 (tt, *J*=7.8, 1.4 Hz, 1H), 8.19–8.26 (m, 2H), 6.03 (s, 2H), 2.29 ppm (s, 3H).

**Preparation of 4‐(4‐bromophenyl)‐6,6′‐dimethyl‐2,2′‐bipyridine (Brdmbpy)**: (*E*)‐3‐(*para*‐Bromophenyl)‐1‐(6‐methyl‐2‐pyridyl)prop‐2‐en‐1‐one (2.00 g, 6.60 mmol, 1,0 equiv.) was dissolved in methanol (90 mL) and 1‐(2‐oxopropyl)pyridinium chloride (1.14 g, 6.60 mmol, 1.0 equiv.) was added. The reaction mixture was treated with ammonium acetate (15.3 g, 198 mmol, 30 equiv.) and refluxed for 24 h. It was left to cool down and put into the fridge overnight. A precipitate formed, which was filtered and washed with water and methanol (3 x 15 mL each). After drying in the desiccator overnight, 4‐(4‐bromophenyl)‐6,6′‐dimethyl‐2,2′‐bipyridine (Brdmbpy) was isolated as a light brown solid (830 mg, 2.44 mmol, 37 %). ^1^H NMR (CDCl_3_, 500 MHz): *δ*=8.45 (s, 1H), 8.27 (d, *J*=7.8 Hz, 1H), 7.74 (t, *J*=7.7 Hz, 1H), 7.65 (d, *J*=1.3 Hz, 4H), 7.37 (d, *J*=1.8 Hz, 1H), 7.21 (d, *J*=7.6 Hz, 1H), 2.72 (s, 3H), 2.68 (s, 3H), 1.98 ppm (s, 1H). ^13^C NMR (500 MHz, CDCl_3_) *δ*/: 158.7, 158.1, 156.2, 155.1, 148.8, 137.7, 137.6, 132.2, 129.0, 123.8, 123.6, 121.1, 119.0, 116.7, 24.7, 24.6 ppm. MALDI‐MS *m*/*z*: 339.1, 341.1 [*M*+H]^+^ (calcd: 339.04, 341.04).

**Preparation of the complex [Ag(dmphen)_2_][PF_6_]**: A solution of the silver(I) hexafluorophosphate (28.1 mg, 0.111 mmol, 1 equiv.) in 5 mL acetonitrile was added into a solution of the 2,9‐dimethyl‐1,10‐phenanthroline (dmphen: 48 mg, 0.221 mmol, 2 equiv.) in 8 mL acetonitrile. The reaction mixture was mixed for 1–2 h before 30 mL diethyl ether was added to the colorless solution. The white complex of [Ag(dmphen)_2_][PF_6_] was precipitated, separated by centrifugation (5 min, 9000 rpm) and dried under vacuum to isolate 68 mg of the [Ag(dmphen)_2_][PF_6_] complex (0.102 mmol, 91.5 %). Slow diffusion of diethyl ether into the solution of the complex in acetonitrile resulted in single crystals of the complexes, which were suitable for X‐ray diffraction studies (Table S1). ^1^H NMR (CD_3_CN, 500 MHz): *δ*=8.57 (d *J*=8.3 Hz, 2H), 8.08 (s, 2H), 7.85 (d, *J*=8.3 Hz, 2H), 2.75 ppm (s, 6H). ^13^C NMR (CD_3_CN, 500 MHz): *δ*=159.8, 142.6, 139.1, 128.1, 126.7, 125.5, 27.3 ppm. MALDI MS: *m*/*z* 523.05, 525.05 [*M*−PF_6_]^+^ (base peak, calcd. 523.11, 525.11). EA: C_28_H_24_F_6_N_4_AgP requires C 50.24, H 3.61, N 8.37 %; found C 50.30, H 3.52, N 8.48 %.

**Preparation of the complex [Ag(Brdmbpy)CH_3_CN][PF_6_]**: A solution of the 4‐(4‐bromophenyl)‐6,6′‐dimethyl‐2,2′‐bipyridine (bpdmbpy, **L5**; 26.8 mg, 0.0791 mmol, 1 equiv.) in 5 mL of dichloromethane was added into a solution of silver(I) hexafluorophosphate (20 mg, 0.0791 mmol, 1 equiv.) in 8 mL dichloromethane. The reaction was mixed for 2 h. The mixture was then concentrated by rotavapor before 20–30 mL of the diethyl ether was added to the solution. The white complex was precipitated and then the product was separated by centrifugation (8 min, 9000 rpm) and dried under vacuum to isolate. Slow diffusion of diethyl ether into the solution of the complex in acetonitrile resulted in single crystals of the complexes 40 mg of the [Ag(Brdmbpy)CH_3_CN][PF_6_] complex (0.063 mmol, 80 %), which were suitable for X‐ray diffraction studies (Table S7). ^1^H NMR (CD_3_CN, 500 MHz): *δ*=8.39 (d, *J*=1.5 Hz, 1H), 8.33 (d, *J*=8.0 Hz, 1H), 8.05 (t, *J*=7.9 Hz, 1H), 7.86–7.75 (m, 5H), 7.58 (d, *J*=7.7 Hz, 1H), 2.72 (s, 3H), 2.69 ppm (s, 3H). MALDI‐MS: *m*/*z* 446.9 [*M*−CH_3_CN−PF_6_]^+^ (base peak, calcd. 446.95).

**Preparation of the complex [Ag(Brdmbpy)_2_][PF_6_]**: A solution of the silver(I) hexafluorophosphate (20.1 mg, 0.0795 mmol, 1 equiv.) in 5 mL of acetonitrile was added into a solution of the 4‐(4‐bromophenyl)‐6,6′‐dimethyl‐2,2′‐bipyridine (Brdmbpy: 54 mg, 0.159 mmol, 2 equiv.) in 12 mL of acetonitrile. The reaction was mixed for 2 h; and 30 mL diethyl ether was then added to the colorless solution. The white precipitate was separated by centrifugation (5 min, 9000 rpm) and dried under vacuum to isolate 70 mg of the complex [Ag(Brdmbpy)_2_][PF_6_] (0.075 mmol, 94.5 %). Slow diffusion of diethyl ether into the solution of the complex in acetonitrile resulted in single crystals of the complexes, which were suitable for X‐ray diffraction studies (Table S4). ^1^H NMR (CD_3_CN, 500 MHz): *δ*=8.44 (d, *J*=1.3 Hz, 1H), 8.36 (d, *J*=8.0 Hz, 1H), 8.03 (t, *J*=7.9 Hz, 1H), 7.84–7.77 (m, 5H), 7.53 (d, *J*=7.6 Hz, 1H), 2.62 (s, 3H), 2.58 ppm (s, 3H). MALDI‐MS: *m*/*z* 784.80 [*M*−PF_6_]^+^ (base peak, calcd. 784.99). C_36_H_30_AgBr_2_F_6_N_4_ requires C 46.43, H 3.25, N 6.02 %; found C 46.51, H 3.69, N 6.06 %.

**Spectroscopic and analytical measurements**: ^1^H NMR and ^13^C{^1^H} NMR spectra were measured at 298 K on a Bruker Avance III‐500 NMR spectrometer. ^1^H and ^13^C chemical shifts were referenced to residual solvent peaks with respect to *δ*(TMS)=0 ppm for ^1^H and ^13^C{^1^H}. Solution absorption spectra were recorded on an Agilent Cary 5000 spectrophotometer. Infrared spectra were recorded on a Perkin Elmer UATR Two spectrometer. MALDI mass spectra were measured using a Shimadzu MALDI‐8020 with α‐cyano‐4‐hydroxycinnamic acid (CHCA) solution as matrix for sample preparation. Spectrophotometric titrations were performed with a J&M diode array spectrometer (Tidas series) connected to an external computer. In a typical experiment, 20 cm^3^ of ligand in acetonitrile (2×10^−4^ M) were titrated at 293 K with a solution of silver salt (10^−3^ M) in acetonitrile under an inert atmosphere. After each addition of 0.1 mL, the absorbance was recorded using Hellma optrodes (optical path length 0.1 cm) immersed in the thermostated titration vessel and connected to the spectrometer. Mathematical treatment of the spectrophotometric titrations was performed with factor analysis[^68^, ^69^, ^70^, ^71^] and with the SPECFIT program.[^72^, ^73^]

**X‐ray crystallography**: Summary of crystal data, intensity measurements and structure refinements were collected in Tables S2, S5, and S8. Single crystal data were collected on a Bruker APEX‐II diffractometer (Cu_Kα_ radiation) with data reduction, solution, and refinement using the programs APEX, ShelXT, Olex2, and ShelXL v. 2014/7, or using a STOE StadiVari diffractometer equipped with a Pilatus300 K detector and with a Metaljet D2 source (GaKα radiation) and solving the structure using Superflip, and Olex2. The structural model was refined with ShelXL v. 2014/7. Structure analysis used Mercury. Deposition Numbers 2150180 (for [Ag(Brdmbpy)_2_][PF_6_]), 2150181 (for [Ag(dmphen)_2_][PF_6_]), and 2150182 (for [Ag(Brdmbpy)CH_3_CN][PF_6_]) contain the supplementary crystallographic data for this paper. These data are provided free of charge by the joint Cambridge Crystallographic Data Centre and Fachinformationszentrum Karlsruhe Access Structures service.

### Supporting Information

Site binding model and statistical factors (Appendices 1–3), detailed X‐ray crystallographic data, spectrophotometric titrations and ^1^H NMR titrations are provided in the Supporting Information.

## Conflict of interest

The authors declare no conflict of interest.

1

## Supporting information

As a service to our authors and readers, this journal provides supporting information supplied by the authors. Such materials are peer reviewed and may be re‐organized for online delivery, but are not copy‐edited or typeset. Technical support issues arising from supporting information (other than missing files) should be addressed to the authors.

Supporting Information

## Data Availability

The data that support the findings of this study are available from the corresponding author upon reasonable request.
