# Supplementary material for: Positive Cooperativity Induced by Interstrand Interactions in Silver(I) Complexes with α,α′‐Diimine Ligands
Source: Chemistry. 2022 Jun 21;28(42):e202200912. doi: 10.1002/chem.202200912 (PMC9401079; doi:10.1002/chem.202200912)
Supplement: Supplementary file 1 — Supporting Information [file CHEM-28-0-s001.pdf]

Chemistry–A European Journal

Supporting Information

Positive Cooperativity Induced by Interstrand Interactions in Silver(I) Complexes with α,α′-Diimine Ligands

Davood Zare,* Claude Piguet,* Alessandro Prescimone, Catherine E. Housecroft, and Edwin C. Constable*

Supporting Information

(23 pages)

Appendix 1 Determination of the thermodynamic descriptors for the formation of $[Cu(L)_n]^+$ complexes in acetonitrile (L = α,α'-diimine ligands collected in Scheme S1).

$$Cu^+_{solvated} + \mathbf{L} \rightleftharpoons [Cu\mathbf{L}]^+_{solvated} \qquad \beta_{1,1}^{Cu,L} = \omega_{1,1}^{Cu,L} f_{Cu}^{L} \tag{1}$$

$$Cu^+_{solvated} + 2\,\mathbf{L} \rightleftharpoons [Cu\mathbf{L}_2]^+_{solvated} \qquad \beta_{1,2}^{Cu,L} = \omega_{1,2}^{Cu,L} \left(f_{Cu}^{L}\right)^2 u_{Cu}^{L\text{-}L} \tag{2}$$

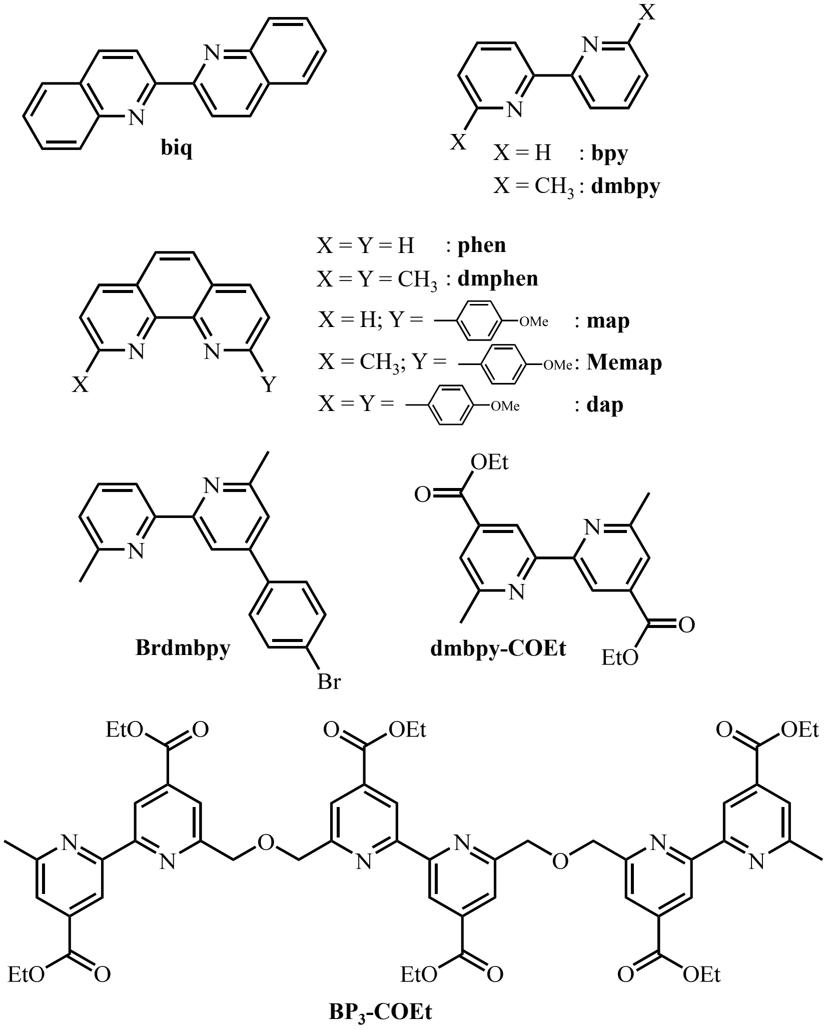

Scheme S1. Chemical structures and acronyms used for the chelating α,α'-diimine ligands considered in Table S1.

Table S1 Thermodynamic formation constants and associated microscopic parameters $\Delta G_{\mathrm{aff}}^{\mathrm{M,L}}$ and $\Delta E_{\mathrm{M}}^{\mathrm{L\text{-}L}}$ obtained for the spectrophotometric titrations of **L** with $[Cu(CH_3CN)_4][BF_4]$ at 298 K.[a]

Ligand	Metal	$\log\left(\beta_{1,1}^{\mathrm{Cu,L}}\right)$	$\log\left(\beta_{1,2}^{\mathrm{Cu,L}}\right)$	$\Delta G_{\mathrm{aff}}^{\mathrm{Cu,L}}$ [b] kJ mol^{-1}	$\Delta E_{\mathrm{Cu}}^{\mathrm{L\text{-}L}}$ [b] kJ mol^{-1}	Solvent	Ref
**biq**	Cu(I)	4.4(1)	8.8(1)	–18.9(4)	–6.2(4)	MeCN + 0.1 M NEt_4ClO_4	[A3]
**bpy**	Cu(I)	3.69(8)	6.5(1)	–14.9(3)	–1.1(1)	MeCN + 0.1 M NEt_4ClO_4	[A1]
**dmbpy**	Cu(I)	5.4(2)	10.2(3)	–24(1)	–3.0(2)	MeCN + 0.1 M NEt_4ClO_4	[A1]
**phen**	Cu(I)	5.2(1)	9.7(1)	–23.5(4)	–2.2(5)	MeCN + 0.1 M NEt_4ClO_4	[A2]
**dmphen**	Cu(I)	6.6(2)	12.3(3)	–31.5(6)	–1.0(1)	MeCN + 0.1 M NBu_4ClO_4	[A2]
**map**	Cu(I)	5.3(2)	11.3(3)	–24.1(5)	–10.2(5)	MeCN + 0.1 M NBu_4ClO_4	[A2]
**Memap**	Cu(I)	5.0(1)	10.7(3)	–22.4(5)	–10.2(5)	MeCN + 0.1 M NBu_4ClO_4	[A2]
**dap**	Cu(I)	4.7(2)	10.7(3)	–20.7(5)	–13.6(7)	MeCN + 0.1 M NBu_4ClO_4	[A2]
**dmbpy-COEt**	Cu(I)	4.5(2)	8.6(4)	–20(1)	–3.9(2)	MeCN/CH_2Cl_2 (1:1)	[A1]
**BP_3-COEt**	Cu(I)	4.6(2)	8.2(3)	–17.4(9)	–0.5(2)	MeCN/CH_2Cl_2/H_2O (85:15:5)	[A1]

[a] The site binding model used $\beta_{1,1}^{\mathrm{M,L}} = 12 f_{\mathrm{M}}^{\mathrm{L}}$ and $\beta_{1,2}^{\mathrm{M,L}} = 12\left(f_{\mathrm{M}}^{\mathrm{L}}\right)^2 u_{\mathrm{M}}^{\mathrm{L\text{-}L}}$ for all ligands except L = BP_3-COEt, for which $\beta_{1,1}^{\mathrm{M,L}} = 36 f_{\mathrm{M}}^{\mathrm{L}}$ and $\beta_{1,2}^{\mathrm{M,L}} = 108\left(f_{\mathrm{M}}^{\mathrm{L}}\right)^2 u_{\mathrm{M}}^{\mathrm{L\text{-}L}}$ is pertinent (see Appendix 1). [b] $\Delta G_{\mathrm{aff}}^{\mathrm{M,L}} = -RT\ln\left(f_{\mathrm{M}}^{\mathrm{L}}\right)$ and $\Delta E_{\mathrm{M}}^{\mathrm{L\text{-}L}} = -RT\ln\left(u_{\mathrm{M}}^{\mathrm{L\text{-}L}}\right)$.

According to Benson,[A4],[A5] the observed equilibrium constant, β, of a generic equilibrium can be regarded as the product of an intrinsic or "chemical" constant β_{chem} and a statistical factor, $\omega_{m,n}^{\text{M,L}}$, given by the ratio of symmetry numbers σ_{tot} for reactant and product species involved in the equilibrium leading to the final metallosupramolecular complex [M_mL_n] (Eq. A1-1).

$$\omega_{m,n}^{\text{M,L}} = \frac{\left(\sigma_{\text{tot}}^{\text{M}}\right)^m \left(\sigma_{\text{tot}}^{\text{L}}\right)^n}{\left(\sigma_{\text{tot}}^{\text{M}_m\text{L}_n}\right)} \quad \text{(A1-1)}$$

Once the point groups of each partner contributing to the equilibrium are known, $\omega_{m,n}^{\text{M,L}}$ can be easily calculated for any M/L couple to give, for instance, $\omega_{1,1}^{\text{Cu,L}}$ = 12 and $\omega_{1,2}^{\text{Cu,L}}$ = 12 for the bidentate α,α'-diimine ligands collected in Scheme S1 and involved in Equilibria (1)-(2) (Appendix 2 and Table 1).[A6] According to the site binding model,[A7] derived from the Potts-Ising model,[A8],[A9] the free energy change accompanying the chemical part of the formation constants $\beta_{\text{chem}} = \beta_{1,n}^{\text{Cu,L}} / \omega_{1,n}^{\text{Cu,L}}$ (Eqs. 1-2) can be partitioned between the intermolecular affinity (including solvation effects) accompanying the complexation of Cu^+ to a single ligand L ($\Delta G_{\text{aff}}^{\text{Cu,L}} = -RT\ln(f_{\text{Cu}}^{\text{L}})$), and the modulation of the latter affinity due to successive ligand binding in $[Cu\mathbf{L}_n]^+$ ($n \geq 2$); a thermodynamic correction $\Delta E_{\text{Cu}}^{\text{L-L}} = -RT\ln(u_{\text{Cu}}^{\text{L-L}})$ often referred to as allosteric cooperativity. Solving the straightforward mathematical model summarized in Eqs. 1-2 for each set of experimental formation constants collected in Table S1 (columns 3-4) provides (i) Cu(I)- α,α'-diimine affinities in the $-32 \leq \Delta G_{\text{aff}}^{\text{Cu,L}} = -RT\ln(f_{\text{Cu}}^{\text{L}}) \leq -15$ kJ mol^{-1} range (column 5) and (ii) systematic positive allosteric cooperativities in the $-14 \leq \Delta E_{\text{Cu}}^{\text{L-L}} = -RT\ln(u_{\text{Cu}}^{\text{L-L}}) \leq -0.5$ kJ mol^{-1} domain (column 6).

References

[A1] N. Fatin-Rouge, S. Blanc, A. Pfeil, A. Rigault, A.-M. Albrecht-Gary; J.-M. Lehn, *Helv. Chim. Acta* **2001**, *84*, 1694-1711.

[A2] M. Meyer, A.-M. Albrecht-Gary, C. O. Dietrich-Buchecker, J.-P. Sauvage, *Inorg. Chem.* **1999**, *38*, 2279-2287.

[A3] U. M. Frei, G. Geier, *Inorg. Chem.* **1992**, *31*, 3132-3137.

[A4] S. W. Benson, *J. Am. Chem. Soc.* **1958**, *80*, 5151-5154.

[A5] W. F. Bailey, A. S. Monahan, *J. Chem. Educ.* **1978**, *55*, 489-493.

[A6] G. Ercolani, C. Piguet, M. Borkovec, J. Hamacek, *J. Phys. Chem. B* **2007**, *111*, 12195-12203.

[A7] C. Piguet, *Chem. Commun.* **2010**, *46*, 6209-6231.

[A8] G. Koper, M. Borkovec, *J. Phys. Chem. B* **2001**, *105*, 6666-6674.

[A9] J. Hamacek, M. Borkovec, C. Piguet, *Dalton Trans.* **2006**, 1473-1490.

Appendix 2 Determination of the statistical factors for the $[Cu(L)_n]^+$ complexes in acetonitrile (L = α,α'-diimine ligands collected in Scheme S1).

The statistical factor $\omega_{m,n}^{\mathrm{M,L}}$ can be estimated by the ratio of symmetry numbers σ_{tot} of the reactant and product species (eqn A2-2) contributing to the equilibrium A2-1.

$$m\,\mathrm{M} + n\,\mathbf{L} \rightleftharpoons [\mathrm{M}_m\mathbf{L}_n] \qquad \beta_{m,n}^{\mathrm{M,L}} = \omega_{m,n}^{\mathrm{M,L}}\beta_{\mathrm{chem}} \qquad \text{(A2-1)}$$

$$\omega_{m,n}^{\mathrm{M,L}} = \frac{\left(\sigma_{\mathrm{tot}}^{\mathrm{M}}\right)^m \left(\sigma_{\mathrm{tot}}^{\mathrm{L}}\right)^n}{\left(\sigma_{\mathrm{tot}}^{\mathrm{M}_m\mathrm{L}_n}\right)} \qquad \text{(A2-2)}$$

Eqn A2-2 is derived from the fact that the symmetry number of a molecule, σ, affects its rotational entropy by a factor of $-R\cdot\ln(\sigma)$. The factor $\sigma_{\mathrm{tot}} = \sigma_{\mathrm{ext}}\sigma_{\mathrm{int}}\sigma_{\mathrm{chir}}$ is the product of the external (σ_{ext}) and internal (σ_{int}) symmetry numbers. The σ_{ext} number is defined as the number of different but indistinguishable atomic arrangements obtained by rotating a given molecule as a whole, while σ_{int} obeys the same definition, but for internal rotation axis. In practice, σ_{ext} is found by multiplying the orders of the independent simple rotational axes of the point group to which the molecule belongs.[46] If a species is chiral and presents as a racemic mixture, the symmetry number must be multiplied by σ_{chir} = ½ to account for the entropy of mixing of the two enantiomers. Applied to the formation of a supramolecular complex made up of two building blocks (eqn A2-1 with M= metal, L= ligand), $\omega_{m,n}^{\mathrm{M,L}}$ takes into account the pure statistical contribution due to the change in the molecular rotational degeneracies occurring when the reactants are transformed into products.

a) **L** = biq, bpy, dmbpy, phen, dmphen, dap, dmbpy-COEt

	$[Cu(CH_3CN)_4]^+$	+ **L** $\xrightarrow{\beta_{1,1}^{\mathrm{Cu,L}}}$	$[Cu(\mathbf{L})(CH_3CN)_2]^+$	+ 2 CH_3CN
Point group	T_d	C_{2h}	C_{2v}	C_{3v}
σ_{ext}	12	2	2	3
σ_{int}	3^4	x[a]	3^2x	1
σ_{chir}	1	1	1	1

$$\omega_{1,1}^{\mathrm{Cu,L}} = \frac{\left(12\cdot3^4\cdot1\right)\cdot\left(2\cdot x\cdot1\right)}{\left(2\cdot3^2\cdot x\cdot1\right)\cdot\left(3\cdot1\cdot1\right)^2} = 12 \Rightarrow \beta_{1,1}^{\mathrm{Cu,L}} = \omega_{1,1}^{\mathrm{Cu,L}} f_{\mathrm{Cu}}^{\mathrm{L}} = 12 f_{\mathrm{Cu}}^{\mathrm{L}}$$

	$[Cu(CH_3CN)_4]^+$	+ 2 **L** $\xrightarrow{\beta_{1,2}^{\mathrm{Cu,L}}}$	$[Cu\mathbf{L}_2]^+$ +	4 CH_3CN
Point group	T_d	C_{2h}	D_{2d}	C_{3v}
σ_{ext}	12	2	4	3
σ_{int}	3^4	x[a]	x^2	1
σ_{chir}	1	1	1	1

$$\omega_{1,2}^{\mathrm{Cu,L}} = \frac{\left(12\cdot3^4\cdot1\right)\cdot\left(2\cdot x\cdot1\right)^2}{\left(4\cdot x^2\cdot1\right)\cdot\left(3\cdot1\cdot1\right)^4} = 12 \Rightarrow \beta_{1,2}^{\mathrm{Cu,L}} = \omega_{1,2}^{\mathrm{Cu,L}}\left(f_{\mathrm{Cu}}^{\mathrm{L}}\right)^2 u_{\mathrm{Cu}}^{\mathrm{L\text{-}L}} = 12\left(f_{\mathrm{Cu}}^{\mathrm{L}}\right)^2 u_{\mathrm{Cu}}^{\mathrm{L\text{-}L}}$$

[a] $x = 1$ for **L** = biq, bpy and phen; $x = 3^2$ for **L** = dmbpy and dmphen; $x = 3^4$ for **L** = dmbpy-COEt and $x = 2^2 3^2$ for **L** = dmbpy-COEt.

b) **L** = map, Memap.

$$[Cu(CH_3CN)_4]^+ + \mathbf{L} \xrightleftharpoons{\beta_{1,1}^{Cu,L}} [Cu(\mathbf{L})(CH_3CN)_2]^+ + 2\ CH_3CN$$

Point group	T_d	C_s	C_s	C_{3v}
σ_{ext}	12	1	1	3
σ_{int}	3^4	x[a]	3^2x	1
σ_{chir}	1	1	1	1

$$\omega_{1,1}^{Cu,L} = \frac{(12\cdot 3^4\cdot 1)\cdot(1\cdot x\cdot 1)}{(1\cdot 3^2\cdot x\cdot 1)\cdot(3\cdot 1\cdot 1)^2} = 12 \quad \Rightarrow \quad \beta_{1,1}^{Cu,L} = \omega_{1,1}^{Cu,L} f_{Cu}^{L} = 12 f_{Cu}^{L}$$

$$[Cu(CH_3CN)_4]^+ + 2\ \mathbf{L} \xrightleftharpoons{\beta_{1,2}^{Cu,L}} [Cu\mathbf{L}_2]^+ + 4\ CH_3CN$$

Point group	T_d	C_s	C_2	C_{3v}
σ_{ext}	12	1	2	3
σ_{int}	3^4	x[a]	x^2	1
σ_{chir}	1	1	½	1

$$\omega_{1,2}^{Cu,L} = \frac{(12\cdot 3^4\cdot 1)\cdot(1\cdot x\cdot 1)^2}{(2\cdot x^2\cdot \tfrac{1}{2})\cdot(3\cdot 1\cdot 1)^4} = 12 \quad \Rightarrow \quad \beta_{1,2}^{Cu,L} = \omega_{1,2}^{Cu,L} \left(f_{Cu}^{L}\right)^2 u_{Cu}^{L\text{-}L} = 12\left(f_{Cu}^{L}\right)^2 u_{Cu}^{L\text{-}L}$$

[a] $x = 2\cdot 3$ for **L** = map; $x = 2\cdot 3^2$ for **L** = Memap.

c) **L** = BP_3-COEt.

Microspecies 1

$$[Cu(CH_3CN)_4]^+ + \mathbf{L} \xrightleftharpoons{\beta_{1,1}^{Cu,L}} \text{T-}[Cu(\mathbf{L})(CH_3CN)_2]^+ + 2\ CH_3CN$$

Point group	T_d	C_{2v}	C_s	C_{3v}
σ_{ext}	12	2	1	3
σ_{int}	3^4	3^8	3^{10}	1
σ_{chir}	1	1	1	1

$$\omega_{1,1}^{Cu,L} = \frac{(12\cdot 3^4\cdot 1)\cdot(2\cdot 3^8\cdot 1)}{(1\cdot 3^{10}\cdot 1)\cdot(3\cdot 1\cdot 1)^2} = 24 \quad \Rightarrow \quad \beta_{1,1}^{Cu,L} = \omega_{1,1}^{Cu,L} f_{Cu}^{L} = 24 f_{Cu}^{L}$$

Microspecies 2

$$[Cu(CH_3CN)_4]^+ + \mathbf{L} \xrightleftharpoons{\beta_{1,1}^{Cu,L}} \text{C-}[Cu(\mathbf{L})(CH_3CN)_2]^+ + 2\ CH_3CN$$

Point group	T_d	C_{2v}	C_{2v}	C_{3v}
σ_{ext}	12	2	2	3
σ_{int}	3^4	3^8	3^{10}	1
σ_{chir}	1	1	1	1

$$\omega_{1,1}^{Cu,L} = \frac{(12\cdot 3^4\cdot 1)\cdot(2\cdot 3^8\cdot 1)}{(2\cdot 3^{10}\cdot 1)\cdot(3\cdot 1\cdot 1)^2} = 12 \quad \Rightarrow \quad \beta_{1,1}^{Cu,L} = \omega_{1,1}^{Cu,L} f_{Cu}^{L} = 12 f_{Cu}^{L}$$

Macrospecies

$$\beta_{1,1}^{Cu,L} = 36 f_{Cu}^{L}$$

Microspecies 1

$$[Cu(CH_3CN)_4]^+ + 2\ \mathbf{L} \xrightleftharpoons{\beta_{1,2}^{Cu,L}} \text{TT-}[Cu\mathbf{L}_2]^+ + 4\ CH_3CN$$

Point group	T_d	C_{2v}	C_2	C_{3v}
σ_{ext}	12	2	2	3
σ_{int}	3^4	3^8	3^{16}	1
σ_{chir}	1	1	½	1

$$\omega_{1,2}^{Cu,L} = \frac{\left(12\cdot 3^4 \cdot 1\right)\cdot\left(2\cdot 3^8 \cdot 1\right)^2}{\left(2\cdot 3^{16} \cdot \tfrac{1}{2}\right)\cdot\left(3\cdot 1\cdot 1\right)^4} = 48 \Rightarrow \beta_{1,2}^{Cu,L} = \omega_{1,2}^{Cu,L}\left(f_{Cu}^{L}\right)^2 u_{Cu}^{L\text{-}L} = 48\left(f_{Cu}^{L}\right)^2 u_{Cu}^{L\text{-}L}$$

Microspecies 2

$$[Cu(CH_3CN)_4]^+ + 2\ \mathbf{L} \xrightleftharpoons{\beta_{1,2}^{Cu,L}} \text{TC-}[Cu\mathbf{L}_2]^+ + 4\ CH_3CN$$

Point group	T_d	C_{2v}	C_s	C_{3v}
σ_{ext}	12	2	1	3
σ_{int}	3^4	3^8	3^{16}	1
σ_{chir}	1	1	1	1

$$\omega_{1,2}^{Cu,L} = \frac{\left(12\cdot 3^4 \cdot 1\right)\cdot\left(2\cdot 3^8 \cdot 1\right)^2}{\left(1\cdot 3^{16} \cdot 1\right)\cdot\left(3\cdot 1\cdot 1\right)^4} = 48 \Rightarrow \beta_{1,2}^{Cu,L} = \omega_{1,2}^{Cu,L}\left(f_{Cu}^{L}\right)^2 u_{Cu}^{L\text{-}L} = 48\left(f_{Cu}^{L}\right)^2 u_{Cu}^{L\text{-}L}$$

Microspecies 3

$$[Cu(CH_3CN)_4]^+ + 2\ \mathbf{L} \xrightleftharpoons{\beta_{1,2}^{Cu,L}} \text{CC-}[Cu\mathbf{L}_2]^+ + 4\ CH_3CN$$

Point group	T_d	C_{2v}	D_{2d}	C_{3v}
σ_{ext}	12	2	4	3
σ_{int}	3^4	3^8	3^{16}	1
σ_{chir}	1	1	1	1

$$\omega_{1,2}^{Cu,L} = \frac{\left(12\cdot 3^4 \cdot 1\right)\cdot\left(2\cdot 3^8 \cdot 1\right)^2}{\left(4\cdot 3^{16} \cdot 1\right)\cdot\left(3\cdot 1\cdot 1\right)^4} = 12 \Rightarrow \beta_{1,2}^{Cu,L} = \omega_{1,2}^{Cu,L}\left(f_{Cu}^{L}\right)^2 u_{Cu}^{L\text{-}L} = 12\left(f_{Cu}^{L}\right)^2 u_{Cu}^{L\text{-}L}$$

Macrospecies

$$\beta_{1,2}^{Cu,L} = 108\left(f_{Cu}^{L}\right)^2 u_{Cu}^{L\text{-}L}$$

Appendix 3 Determination of the statistical factors for the $[Ag(L)_n]^+$ complexes in acetonitrile (L = α,α'-diimine ligands collected in Scheme 1).

a) **L** = bpy, dmbpy, phen, dmphen

$$[Ag(CH_3CN)_4]^+ + \mathbf{L} \overset{\beta_{1,1}^{Ag,L}}{\rightleftharpoons} [Ag(\mathbf{L})(CH_3CN)]^+ + 3\ CH_3CN$$

	$[Ag(CH_3CN)_4]^+$	L	$[Ag(L)(CH_3CN)]^+$	3 CH_3CN
Point group	T_d	C_{2h}	C_{2v}	C_{3v}
σ_{ext}	12	2	2	3
σ_{int}	3^4	x[a]	$3x$	1
σ_{chir}	1	1	1	1

$$\omega_{1,1}^{Ag,L} = \frac{\left(12\cdot 3^4\cdot 1\right)\cdot\left(2\cdot x\cdot 1\right)}{\left(2\cdot 3\cdot x\cdot 1\right)\cdot\left(3\cdot 1\cdot 1\right)^3} = 12 \quad \Rightarrow \quad \beta_{1,1}^{Ag,L} = \omega_{1,1}^{Ag,L} f_{Ag}^{L} = 12 f_{Ag}^{L}$$

$$[Ag(CH_3CN)_4]^+ + 2\ \mathbf{L} \overset{\beta_{1,2}^{Ag,L}}{\rightleftharpoons} [Ag\mathbf{L}_2]^+ + 4\ CH_3CN$$

	$[Ag(CH_3CN)_4]^+$	2 L	$[AgL_2]^+$	4 CH_3CN
Point group	T_d	C_{2h}	D_{2d}	C_{3v}
σ_{ext}	12	2	4	3
σ_{int}	3^4	x[a]	x^2	1
σ_{chir}	1	1	1	1

$$\omega_{1,2}^{Ag,L} = \frac{\left(12\cdot 3^4\cdot 1\right)\cdot\left(2\cdot x\cdot 1\right)^2}{\left(4\cdot x^2\cdot 1\right)\cdot\left(3\cdot 1\cdot 1\right)^4} = 12 \quad \Rightarrow \quad \beta_{1,2}^{Ag,L} = \omega_{1,2}^{Ag,L}\left(f_{Ag}^{L}\right)^2 u_{Ag}^{L\text{-}L} = 12\left(f_{Ag}^{L}\right)^2 u_{Ag}^{L\text{-}L}$$

[a] $x = 1$ for **L** = bpy and phen; $x = 3^2$ for **L** = dmbpy and dmphen.

b) **L** = Brdmbpy

$$[Ag(CH_3CN)_4]^+ + \mathbf{L} \overset{\beta_{1,1}^{Ag,L}}{\rightleftharpoons} [Ag(\mathbf{L})(CH_3CN)]^+ + 3\ CH_3CN$$

	$[Ag(CH_3CN)_4]^+$	L	$[Ag(L)(CH_3CN)]^+$	3 CH_3CN
Point group	T_d	C_s	C_s	C_{3v}
σ_{ext}	12	1	1	3
σ_{int}	3^4	$2\cdot 3^2$	$2\cdot 3^3$	1
σ_{chir}	1	1	1	1

$$\omega_{1,1}^{Ag,L} = \frac{\left(12\cdot 3^4\cdot 1\right)\cdot\left(1\cdot 2\cdot 3^2\cdot 1\right)}{\left(1\cdot 2\cdot 3^3\cdot 1\right)\cdot\left(3\cdot 1\cdot 1\right)^3} = 12 \quad \Rightarrow \quad \beta_{1,1}^{Ag,L} = \omega_{1,1}^{Ag,L} f_{Ag}^{L} = 12 f_{Ag}^{L}$$

$$[Ag(CH_3CN)_4]^+ + 2\ \mathbf{L} \overset{\beta_{1,2}^{Ag,L}}{\rightleftharpoons} [Ag\mathbf{L}_2]^+ + 4\ CH_3CN$$

	$[Ag(CH_3CN)_4]^+$	2 L	$[AgL_2]^+$	4 CH_3CN
Point group	T_d	C_s	C_2	C_{3v}
σ_{ext}	12	1	2	3
σ_{int}	3^4	$2\cdot 3^2$	$2^2\cdot 3^4$	1
σ_{chir}	1	1	½	1

$$\omega_{1,2}^{Ag,L} = \frac{\left(12\cdot 3^4\cdot 1\right)\cdot\left(1\cdot 2\cdot 3^2\cdot 1\right)^2}{\left(2\cdot 2^2\cdot 3^4\cdot \tfrac{1}{2}\right)\cdot\left(3\cdot 1\cdot 1\right)^4} = 12 \quad \Rightarrow \quad \beta_{1,2}^{Ag,L} = \omega_{1,2}^{Ag,L}\left(f_{Ag}^{L}\right)^2 u_{Ag}^{L\text{-}L} = 12\left(f_{Ag}^{L}\right)^2 u_{Ag}^{L\text{-}L}$$

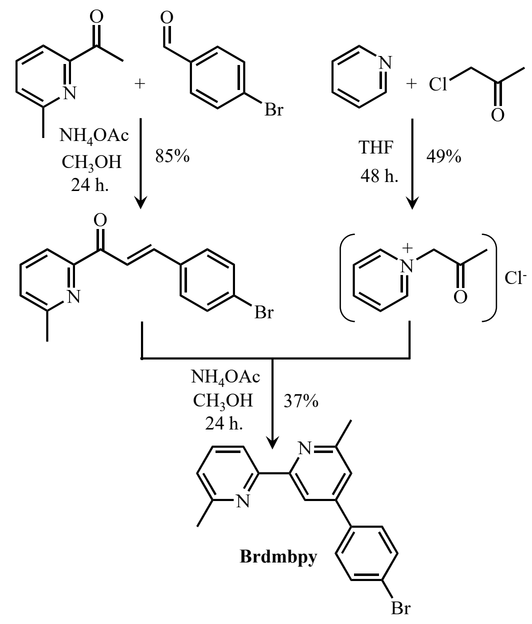

Scheme S2. Synthesis of ligand **Brdmbpy**.

Table S2 Crystal data and structure refinement for complex [Ag(**dmphen**)$_2$][PF_6].

CCDC	2150181	
Empirical formula	$C_{28}H_{24}AgF_6N_4P$	
Formula weight	669.35	
Temperature	123 K	
Wavelength	1.54178 Å	
Crystal system	Monoclinic	
Space group	*P* 21/n	
Unit cell dimensions	a = 10.1121(6) Å	α = 90°
	b = 13.7802(8) Å	β = 91.707(2)°
	c = 19.0436(11) Å	γ = 90°
Volume	2652.5(3) Å^3	
Z	4	
Density (calculated)	1.676 g/cm^3	
Absorption coefficient	7.283 mm^{-1}	
F(000)	1344.0	
Crystal size	0.050 x 0.050 x 0.080 mm^3	
Theta range for data collection	3.960 to 70.396°	
Index ranges	-12<=*h*<=12, -16<=*k*<=16, -23<=*l*<=22	
Reflections collected	18757	
Independent reflections	4911 [*R*(int) = 0.029]	
Completeness to theta = 68°	0.993%	
Absorption correction	Multi-Scan	
Max. and min. transmission	0.520 and 0.690	
Refinement method	Full-matrix least-squares on F^2	
Data / restraints / parameters	4890 / 0 / 361	
Goodness-of-fit on F^2	1.002	
Final *R* indices [*I*>2sigma(*I*)]	*R*1 = 0.0259, w*R*2 = 0.0642	
R indices (all data)	*R*1 = 0.0279, w*R*2 = 0.0645	
Extinction coefficient	n/a	
Largest diff. peak and hole	0.62 and -0.62 e.Å^{-3}	

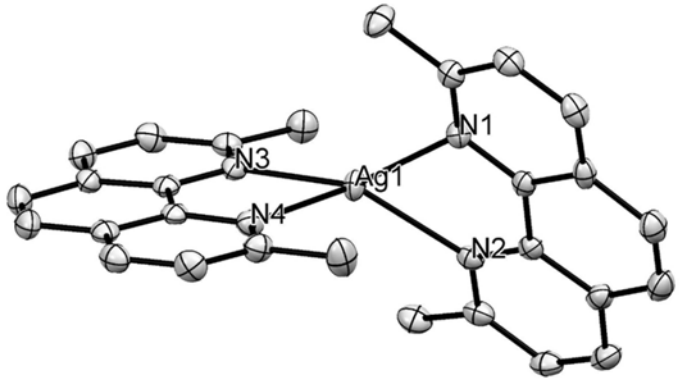

Figure S1 ORTEP view of the [Ag(**dmphen**)$_2$]$^+$ complex with partial numbering scheme taken from the crystal structure of [Ag(**dmphen**)$_2$][PF_6] (thermal ellipsoids are shown at 50% probability level). The solvent molecules, hydrogen atoms and counterions were omitted for clarity.

Table S3 Selected bond distances (Å) and bond angles (°) for complex [Ag(**dmphen**)$_2$][PF_6].

Bond Distances /Å					
Atom	Atom	Length/Å	Atom	Atom	Length/Å
Ag1	N1	2.3228 (17)	Ag1	N3	2.2585 (16)
Ag1	N2	2.3304 (17)	Ag1	N4	2.4243 (17)

Bond Angles (°)			
Atom	Atom	Atom	Angle /°
N1	Ag1	N2	72.31 (6)
N1	Ag1	N3	149.66 (6)
N1	Ag1	N4	105.17 (6)
N2	Ag1	N3	134.18 (6)
N2	Ag1	N4	127.01 (5)
N3	Ag1	N4	72.30 (6)

Table S4 Selected least-squares planes data for complex [Ag(**dmphen**)$_2$][PF_6].

Plane	mean deviation in Å	max deviation in Å (atom)
**Py1**: N1 C4 C6 C8 C9 C10	0.006	0.012 (C6)
**Py2**: N2 C3 C13 C14 C15 C16	0.011	0.018 (N2)
**Py3**: N3 C19 C28 C29 C30 C31	0.001	0.002 (N3)
**Py4**: N4 C20 C22 C23 C24 C25	0.005	0.011 (C20)

Mean and maximum deviation between the atoms of the pyridine rings and their best plane.

Planes	Interplanar angle (°)
Py1-Py2	3.38(6)
Py1-Py3	68.42(6)
Py1-Py4	68.16(6)
Py2-Py3	65.31(6)
Py2-Py4	65.11(6)
Py3-Py4	2.48(6)

Table S5 Crystal data and structure refinement for complex [Ag(**Brdmbpy**)$_2$][PF_6].

CCDC	2150180	
Empirical formula	$C_{36}H_{30}AgBr_2F_6N_4P$	
Formula weight	931.30	
Temperature	123 K	
Wavelength	1.54178 Å	
Crystal system	Triclinic	
Space group	*P* -1	
Unit cell dimensions	a = 1.6569(11) Å	α = 88.559(3)°
	b = 12.5186(12) Å	β = 88.118(3)°
	c = 25.302(2) Å	γ = 73.776(3)°
Volume	3542.8(6) Å^3	
Z	4	
Density (calculated)	1.746 g/cm^3	
Absorption coefficient	8.199 mm^{-1}	
F(000)	1840.0	
Crystal size	0.05 x 0.08 x 0.10 mm^3	
Theta range for data collection	1.747 to 70.310°	
Index ranges	-13<=*h*<=14, -14<=*k*<=14, -30<=*l*<=30	
Reflections collected	44751	
Independent reflections	12693 [*R*(int) = 0.033]	
Completeness to theta = 68°	97.5 %	
Absorption correction	Multi-Scan	
Max. and min. transmission	0.530 and 0.660	
Refinement method	Full-matrix least-squares on F^2	
Data / restraints / parameters	12590 / 0 / 901	
Goodness-of-fit on F^2	0.859	
Final *R* indices [*I*>2sigma(*I*)]	*R*1 = 0.0538, w*R*2 = 0.0.1376	
R indices (all data)	*R*1 = 0.0561, w*R*2 = 0.0.1378	
Extinction coefficient	n/a	
Largest diff. peak and hole	2.00 and -1.65 e.Å^{-3}	

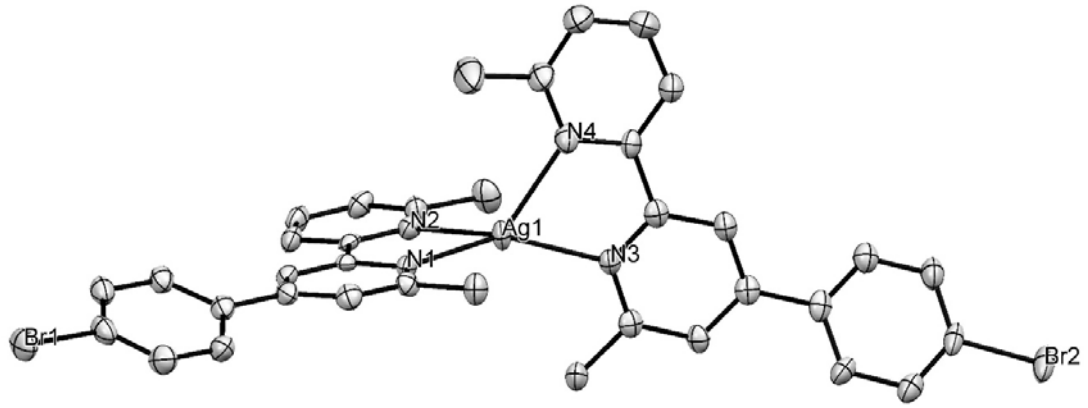

Figure S2 ORTEP view of the [Ag(**Brdmbpy**)$_2$]$^+$ complex with partial numbering scheme taken from the crystal structure of [Ag(**Brdmbpy**)$_2$][PF_6] (thermal ellipsoids are shown at 50% probability level). The solvent molecules, hydrogen atoms and counterions were omitted for clarity.

Table S6 Selected bond distances (Å) and bond angles (°) for complex [Ag(**Brdmbpy**)$_2$][PF_6].

Bond Distances /Å					
Atom	Atom	Length/Å	Atom	Atom	Length/Å
Ag1	N1	2.279 (4)	Ag1	N3	2.276 (4)
Ag1	N2	2.347 (4)	Ag1	N4	2.331 (5)

Bond Angles (°)			
Atom	Atom	Atom	Angle /°
N1	Ag1	N2	71.20 (15)
N1	Ag1	N3	146.31 (15)
N1	Ag1	N4	127.92 (15)
N2	Ag1	N3	127.32 (15)
N2	Ag1	N4	117.84 (16)
N3	Ag1	N4	72.51 (16)

Table S7 Selected least-squares planes data for complex [Ag(**Brdmbpy**)$_2$][PF_6].

Plane	mean deviation in Å	max deviation in Å (atom)
**Py1**: N1 C2 C3 C4 C11 C12	0.009	0.009 (C11)
**Py2**: N2 C13 C14 C15 C16 C17	0.006	0.009 (C17)
**Py3**: N3 C20 C21 C22 C29 C30	0.012	0.019 (C30)
**Py4**: N4 C31 C32 C33 C34 C35	0.004	0.007 (C33)

Mean and maximum deviation between the atoms of the pyridine rings and their best plane.

Planes	Interplanar angle (°)
Py1-Py2	3.11 (5)
Py1-Py3	89.50 (5)
Py1-Py4	79.52 (5)
Py2-Py3	89.92 (5)
Py2-Py4	79.84 (5)
Py3-Py4	19.41 (5)

Table S8 Crystal data and structure refinement for complex [Ag(**Brdmbpy**)CH_3CN][PF_6].

CCDC	2150182	
Empirical formula	$C_{20}H_{18}AgBrF_6N_3P$	
Formula weight	633.12	
Temperature	123 K	
Wavelength	1.54178 Å	
Crystal system	Monoclinic	
Space group	$P2_1/c$	
Unit cell dimensions	*a* = 11.3191(9) Å	α = 90°
	b = 18.4457(14) Å	β = 117.377(2)°
	c = 11.9502(9) Å	γ = 90°
Volume	2215.6(3) Å^3	
Z	4	
Density (calculated)	1.898 g/cm^3	
Absorption coefficient	10687 mm^{-1}	
F(000)	1240.0	
Crystal size	0.050 x 0.050 x 0.090 mm^3	
Theta range for data collection	4.399 to 69.088°	
Index ranges	-13<=*h*<=13, -22<=*k*<=21, -14<=*l*<=14	
Reflections collected	54559	
Independent reflections	4108 [*R*(int) = 0.029]	
Completeness to theta = 68°	100.0 %	
Absorption correction	Multi-Scan	
Max. and min. transmission	0.390 and 0.590	
Refinement method	Full-matrix least-squares on F^2	
Data / restraints / parameters	4088 / 0 / 289	
Goodness-of-fit on F^2	0.905	
Final *R* indices [*I*>2sigma(*I*)]	*R*1 = 0.0224, w*R*2 = 0.0416	
R indices (all data)	*R*1 = 0.0224, w*R*2 = 0.0416	
Extinction coefficient	n/a	
Largest diff. peak and hole	0.38 and -0.36 e.Å^{-3}	

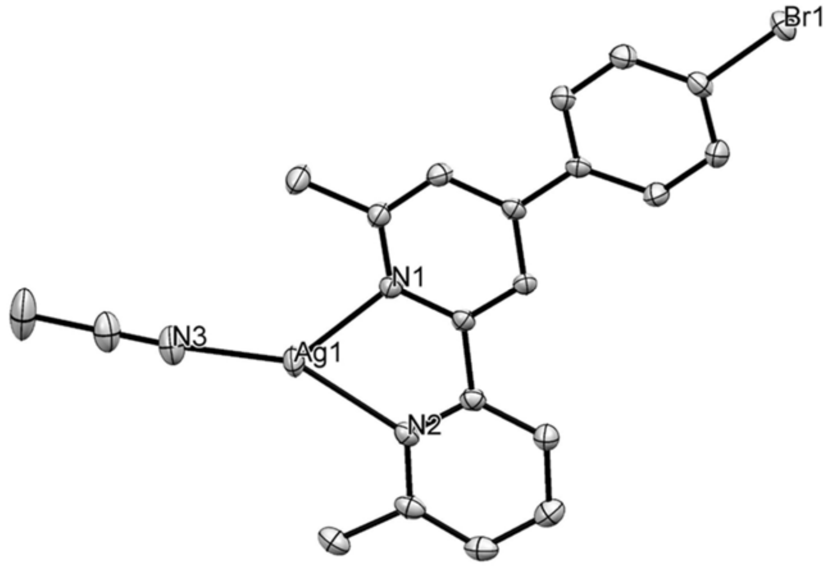

Figure S3 ORTEP view of the [Ag(**Brdmbpy**)CH_3CN]$^+$ complex with partial numbering scheme taken from the crystal structure of [Ag(**Brdmbpy**)CH_3CN][PF_6] (thermal ellipsoids are shown at 50% probability level). The solvent molecules, hydrogen atoms and counterions were omitted for clarity.

Table S9 Selected bond distances (Å) and bond angles (°) for complex [Ag(**Brdmbpy**)CH_3CN][PF_6].

Bond Distances /Å					
Atom	Atom	Length/Å	Atom	Atom	Length/Å
Ag1	N1	2.2852 (15)	Ag1	N3	2.1104 (18)
Ag1	N2	2.2658 (16)			

Bond Angles (°)			
Atom	Atom	Atom	Angle /°
N1	Ag1	N2	73.08 (5)
N1	Ag1	N3	134.74 (7)
N2	Ag1	N3	152.18 (7)

Table S10 Selected least-squares planes data for complex [Ag(**Brdmbpy**)CH_3CN][PF_6].

Plane	mean deviation in Å	max deviation in Å (atom)
**Py1**: N1 C7 C8 C9 C10 C11	0.008	0.014 (C10)
**Py2**: N2 C2 C3 C4 C5 C6	0.009	0.016 (C6)

Mean and maximum deviation between the atoms of the pyridine rings and their best plane.

Planes	Interplanar angle (°)
Py1-Py2	7.87 (7)

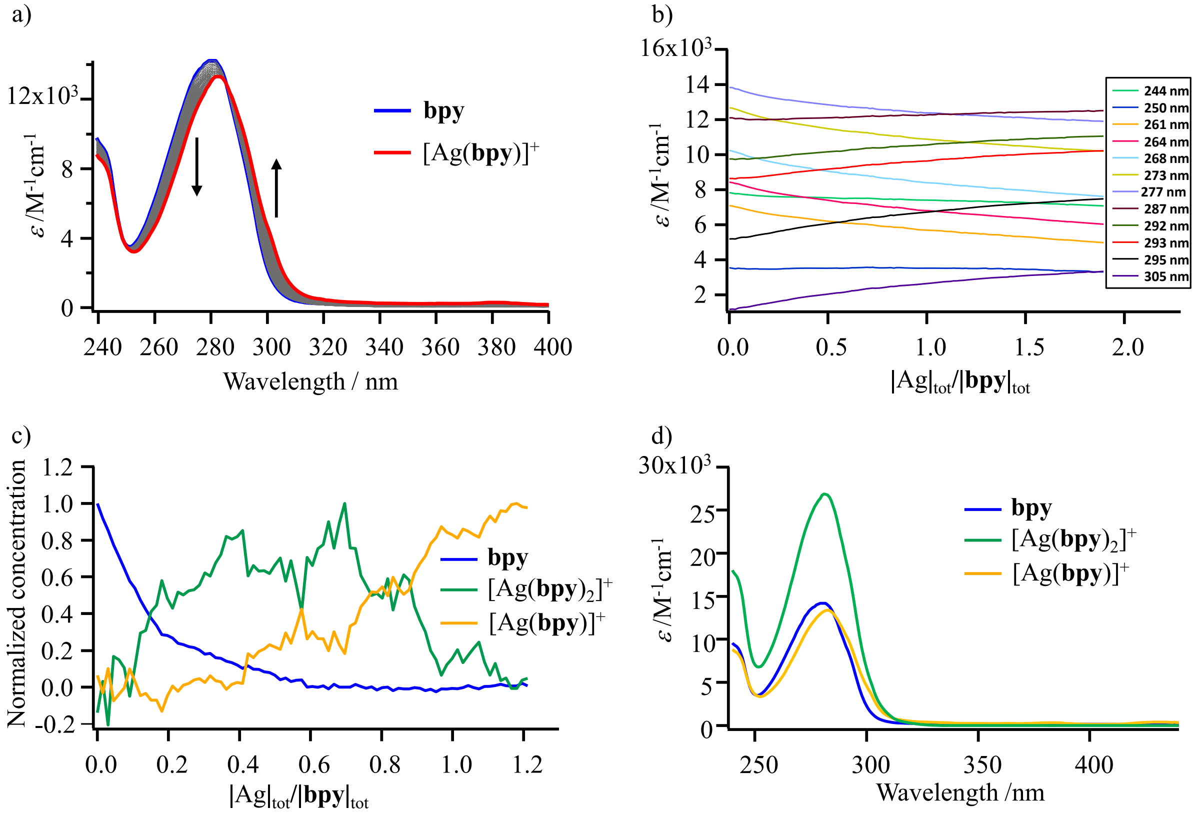

Figure S4. a) Variation of experimental absorption spectra and b) corresponding variation of molar extinction at different wavelengths observed for the spectrophotometric titration of **bpy** with $AgPF_6$ (total ligand concentration: 2.8×10^{-4} M in acetonitrile, 298 K titration increment $|Ag|_{tot}/|\mathbf{bpy}|_{tot} \approx 0.05$). c) Evolving factor analysis [72]-[75] using three absorbing eigenvectors, each normalized to a maximum concentration of 1.0[77] d) reconstructed individual electronic absorption spectra.

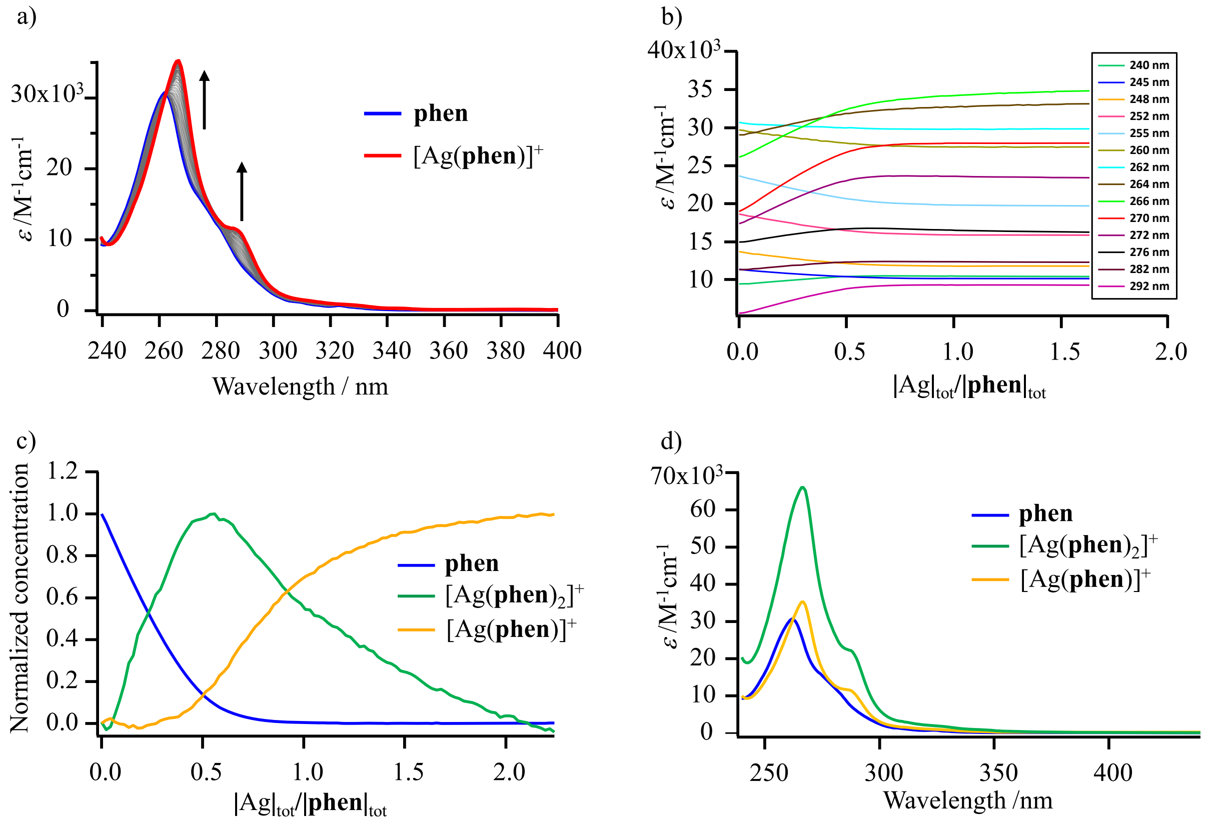

Figure S5. a) Variation of experimental absorption spectra and b) corresponding variation of molar extinction at different wavelengths observed for the spectrophotometric titration of **phen** with $AgPF_6$ (total ligand concentration: 2.1×10^{-4} M in acetonitrile, 298 K (total ligand concentration: 2.8×10^{-4} M in acetonitrile, 298 K titration increment $|\mathrm{Ag}|_{tot}/|\mathbf{phen}|_{tot} \approx 0.05$). c) Evolving factor analysis [72]-[75] using three absorbing eigenvectors, each normalized to a maximum concentration of 1.0[77] d) reconstructed individual electronic absorption spectra.

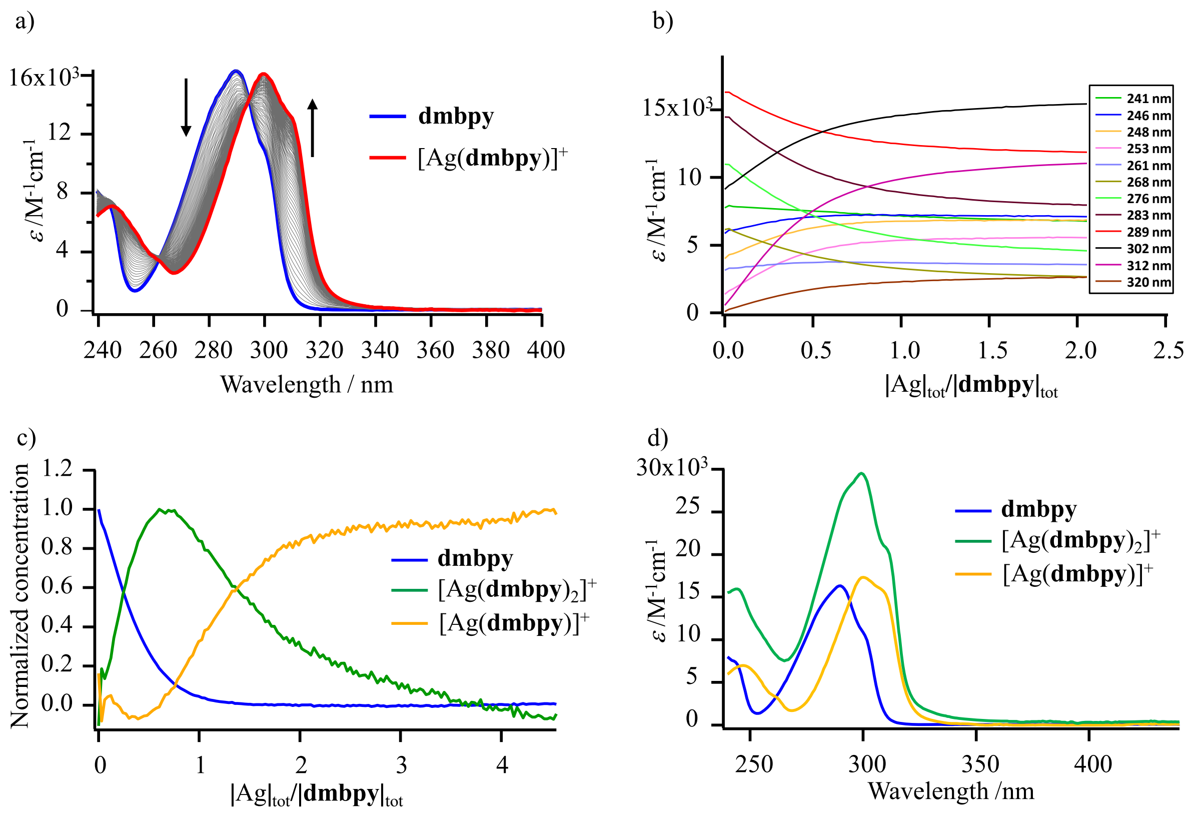

Figure S6. a) Variation of experimental absorption spectra and b) corresponding variation of molar extinction at different wavelengths observed for the spectrophotometric titration of **dmbpy** with $AgPF_6$ (total ligand concentration: 2.5×10^{-4} M in acetonitrile, 298 K (total ligand concentration: 2.8×10^{-4} M in acetonitrile, 298 K titration increment $|Ag|_{tot}/|\mathbf{dmbpy}|_{tot} \approx 0.05$). c) Evolving factor analysis [72]-[75] using three absorbing eigenvectors, each normalized to a maximum concentration of 1.0[77] d) reconstructed individual electronic absorption spectra.

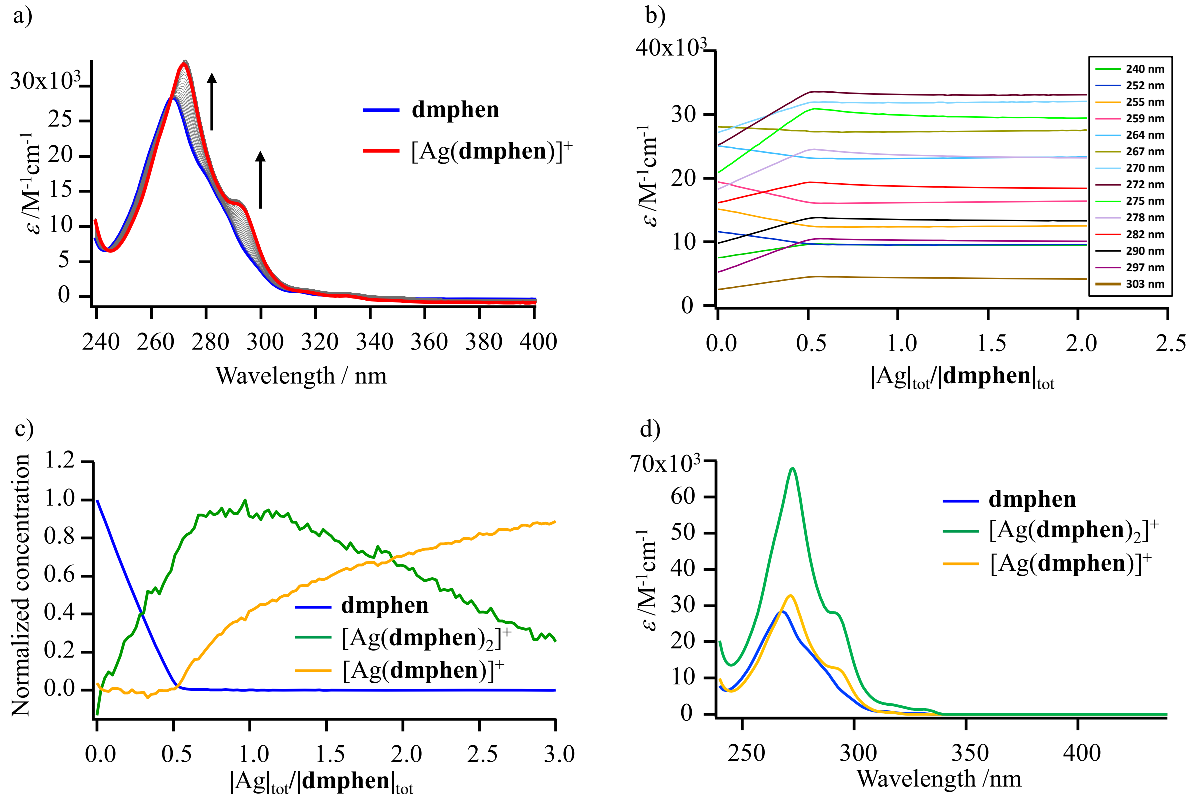

Figure S7. a) Variation of experimental absorption spectra and b) corresponding variation of molar extinction at different wavelengths observed for the spectrophotometric titration of **dmphen** with $AgPF_6$ (total ligand concentration: 2.1×10^{-4} M in acetonitrile, 298 K). (total ligand concentration: 2.8×10^{-4} M in acetonitrile, 298 K titration increment $|Ag|_{tot}/|\mathbf{dmphen}|_{tot} \approx 0.05$). c) Evolving factor analysis [72]-[75] using three absorbing eigenvectors, each normalized to a maximum concentration of 1.0[77] d) reconstructed individual electronic absorption spectra..

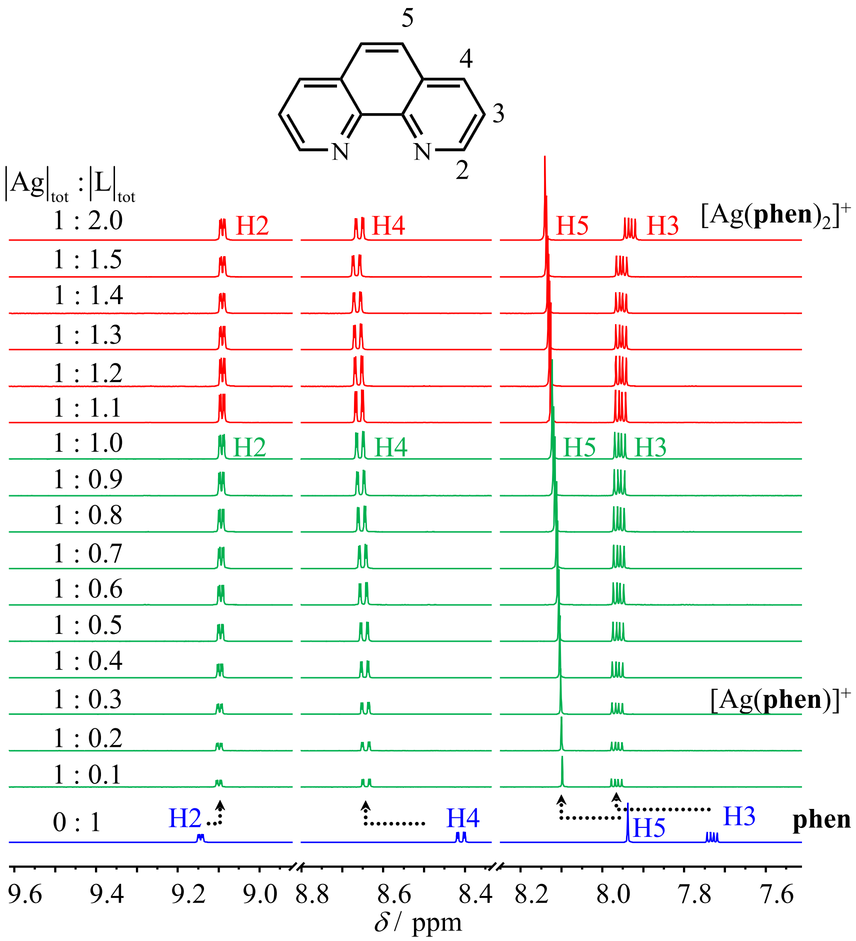

Figure S8. ^{1}H-NMR batch titration of **phen** ($|L|_{tot}$ = 5 mM) with $AgPF_6$ (500 MHz, CD_3CN, 293 K).

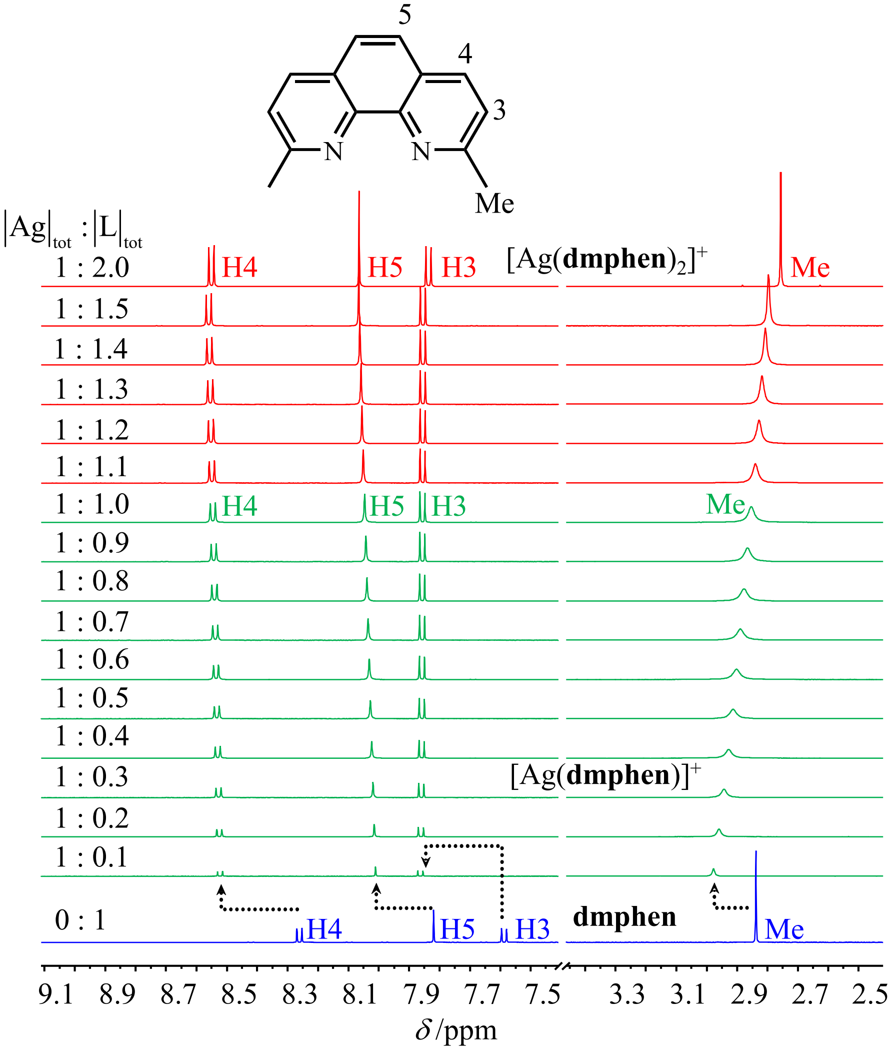

Figure S9. ^{1}H-NMR batch titration of **dmphen** ($|L|_{tot}$ = 5 mM) with $AgPF_6$ (500 MHz, CD_3CN, 293 K).
